# The effectiveness of hand hygiene interventions for preventing community transmission or acquisition of novel coronavirus or influenza infections: a systematic review

**DOI:** 10.1186/s12889-022-13667-y

**Published:** 2022-07-02

**Authors:** Lucyna Gozdzielewska, Claire Kilpatrick, Jacqui Reilly, Sally Stewart, John Butcher, Andrew Kalule, Oliver Cumming, Julie Watson, Lesley Price

**Affiliations:** 1 Department of Nursing and Community Health, Research Centre for Health, Glasgow Caledonian University, Cowcaddens Road, Glasgow, G4 0BA Scotland, UK; 2 KSHealthcare Consulting, 40 Craigiehall Place, Glasgow, G51 1TN UK; 3 Department of Disease Control, Faculty of Infectious Tropical Disease, London School of Hygiene and Tropical Medicine, Keppel Street, London, WC1E 7HT UK

**Keywords:** Hand hygiene, Hand washing, Community transmission, Community acquisition, SARS-CoV-1, SARS-CoV-2, COVID-19, Influenza, Systematic review

## Abstract

**Background:**

Novel coronaviruses and influenza can cause infection, epidemics, and pandemics. Improving hand hygiene (HH) of the general public is recommended for preventing these infections. This systematic review examined the effectiveness of HH interventions for preventing transmission or acquisition of such infections in the community.

**Methods:**

PubMed, MEDLINE, CINAHL and Web of Science databases were searched (January 2002–February 2022) for empirical studies related to HH in the general public and to the acquisition or transmission of novel coronavirus infections or influenza. Studies on healthcare staff, and with outcomes of compliance or absenteeism were excluded. Study selection, data extraction and quality assessment, using the Cochrane Effective Practice and Organization of Care risk of bias criteria or Joanna Briggs Institute Critical Appraisal checklists, were conducted by one reviewer, and double-checked by another. For intervention studies, effect estimates were calculated while the remaining studies were synthesised narratively. The protocol was pre-registered (PROSPERO 2020: CRD42020196525).

**Results:**

Twenty-two studies were included. Six were intervention studies evaluating the effectiveness of HH education and provision of products, or hand washing against influenza. Only two school-based interventions showed a significant protective effect (OR: 0.64; 95% CI 0.51, 0.80 and OR: 0.40; 95% CI 0.22, 0.71), with risk of bias being high (*n* = 1) and unclear (*n* = 1). Of the 16 non-intervention studies, 13 reported the protective effect of HH against influenza, SARS or COVID-19 (*P* < 0.05), but risk of bias was high (*n* = 7), unclear (*n* = 5) or low (*n* = 1). However, evidence in relation to when, and how frequently HH should be performed was inconsistent.

**Conclusions:**

To our knowledge, this is the first systematic review of effectiveness of HH for prevention of community transmission or acquisition of respiratory viruses that have caused epidemics or pandemics, including SARS-CoV-1, SARS-CoV-2 and influenza viruses. The evidence supporting the protective effect of HH was heterogeneous and limited by methodological quality; thus, insufficient to recommend changes to current HH guidelines. Future work is required to identify in what circumstances, how frequently and what product should be used when performing HH in the community and to develop effective interventions for promoting these specific behaviours in communities during epidemics.

**Supplementary Information:**

The online version contains supplementary material available at 10.1186/s12889-022-13667-y.

## Introduction

Novel coronaviruses, emerging from animal reservoirs over the past two decades are a global public health concern as they cause severe illness, epidemics and pandemics. Infections caused by novel coronaviruses include coronavirus disease 2019 (COVID-19), Middle East respiratory syndrome (MERS), and severe acute respiratory syndrome (SARS) [1]. The first novel coronavirus (SARS-CoV-1) emerged in 2002 in China [2], causing over 8000 SARS cases before it was contained in 2003 [3]. In 2012, MERS-CoV virus emerged in Saudi Arabia with more than 2500 MERS confirmed cases to date [4]. These figures are relatively small compared to the current COVID-19 pandemic caused by SARS-CoV-2 virus, with over 509.5 million cases and over 6 million deaths reported worldwide by 25th of April 2022 [5]. COVID-19 is a defining global emergency, challenging healthcare systems, the economy and people’s lives.

Another group of respiratory viruses with capacity to cause pandemics are influenza viruses. The most recent influenza pandemic, caused by an influenza A Hemagglutinin Type 1 and Neuraminidase Type 1 (H1N1) virus, occurred in 2009 and might have caused more than half a million deaths globally within the first year [6]. Yet, the impact of influenza pandemics can be even greater, with the 1918 influenza pandemic estimated to have caused over 50 million deaths [7]. According to the World Health Organization (WHO) influenza and novel coronaviruses cause health, social and economic devastation worldwide [8, 9]. Therefore, there is a necessity to identify effective measures, for limiting the transmission and acquisition of these infections.

Hand hygiene (HH), defined as cleaning hands to reduce the microbial load [10, 11], has been identified as a principal measure for preventing transmission of respiratory diseases [12, 13]. HH can be performed either by hand washing with soap or by handrubbing with alcohol-based hand rub (ABHR). Given initial evidence that the SARS-CoV-2 virus was mainly transmitted via respiratory particles and contact [14], the WHO’s recommendations on HH during the COVID-19 pandemic illustrates the importance of HH for prevention of respiratory infections. In fact, the WHO [14] has advised countries to improve HH practices by providing universal access to public HH stations and making their use mandatory on entering and leaving public or private commercial buildings or public transport facilities. However, they cite no supporting evidence for the effectiveness of HH in reducing the transmission or acquisition of novel coronaviruses in the community. The aim of this systematic review is to synthesise the available evidence regarding the effectiveness of HH and HH interventions for prevention of transmission or acquisition of COVID-19, MERS, SARS or influenza. The following questions guided the review:

1. Is HH effective in preventing the transmission or acquisition of novel coronavirus or influenza infections that have caused epidemics or pandemics?
2. What community HH interventions are effective in preventing the transmission or acquisition of novel coronavirus or influenza infections that have caused epidemics or pandemics?

## Methods

This review was pre-registered on the international prospective register of systematic reviews (PROSPERO) (https://www.crd.york.ac.uk/prospero/display_record.php?RecordID=196525) and is reported in accordance with the Preferred Reporting Items for Systematic Reviews and Meta-Analyses (PRISMA) statement [15].

### Search strategy

PubMed, MEDLINE, and CINAHL electronic databases along with databases on the Web of Science gateway (Core Collection, Current Contents, and KCI databases) were searched using a combination of free text words and index terms or Medical Subject Headings (MeSH) terms within titles and abstracts. Search terms were related to four areas: (1) population – humans; (2) settings – community; (3) intervention/area of interest – HH; and (4) context – COVID-19, SARS, MERS or influenza infections. Because the first novel coronavirus epidemic, caused by SARS-CoV-1 virus, emerged in 2002 [2, 16], the search was restricted to articles published from 2002 to February 2022. We searched for articles published in any language and in any geographical location. The search strategy was adjusted to the functionality of each database. The search strategy applied in MEDLINE is presented in Additional file 1.

In addition, the reference lists of included studies and relevant literature reviews identified through the search were reviewed to identify any additional relevant articles.

### Eligibility criteria & study selection

Identified articles were screened against the eligibility criteria by title and abstract, and subsequently by full text, by one reviewer (LG). Another reviewer (LP, AK or JB) checked these decisions and disagreements were resolved through discussions. For the foreign-language articles, an online translation tool was used to translate the text, while numerical data did not require translation.

All primary research studies, including experimental, quasi-experimental and observational designs, focusing on the public, in the context of community settings, and investigating the effectiveness of HH or the effectiveness of HH interventions for improving HH practices, with individual level outcomes related to acquisition or transmission of confirmed COVID-19, SARS, MERS or influenza, were included in the review. Non-primary research records, articles focusing on healthcare workers, workplaces or with outcomes at the population level or related to serological testing, HH compliance, or school or work absenteeism were excluded.

### Data extraction and quality assessment

One reviewer (LG) extracted data from eligible studies using a standard tool, pre-designed for the review. All extracted data were checked for accuracy by one of five other reviewers (AK, CK, JR, JB, LP or SS). Extracted data included country of origin, study aim, study design, type of infection(s), population, sample, sampling methods, intervention, comparator, intervention fidelity, data collection methods, relevant outcomes, and results.

The risk of bias of randomised controlled trials (RCT) was assessed using standard EPOC risk of bias criteria [17]. Studies were considered as high risk of bias if any of the criteria were assessed as such, unclear risk if there was insufficient information to make a judgement for at least one of the criteria, and low risk if all criteria were assessed as low risk. EPOC risk of bias criteria were designed specifically for RCTs, non-randomised trials, controlled before-after studies and interrupted time series [17]. Remaining study designs were assessed for quality using either the Joanna Briggs Institute’s cross-sectional, case-controlled or cohort study critical appraisal checklists [18]. If an answer to any item on the checklist was “no”, the study was assessed as high risk of bias, if insufficient information was available for any item the study was considered to have an unclear risk of bias. Studies with all checklist items answered as “yes” were classified as low risk of bias. Regardless of the tool used, the quality of all included studies was assessed by one of five reviewers (LG, AK, CK, JR, JB or SS), with all decisions checked by another reviewer (AK, CK, JR, JB, LP or LG). Disagreements were resolved through discussion, or with the involvement of another experienced reviewer (LP). The strengths and limitations of studies are highlighted in the discussion of the results.

### Data analysis

Data from intervention and non-intervention studies were analysed separately. Evidence was further grouped according to the type of infection and population and a narrative synthesis of evidence was carried out.

A meta-analysis was not considered appropriate due to the limited number of intervention studies and high heterogeneity across the interventions, outcomes and settings. Adjusted odds ratios were only reported in one [19] of six intervention studies and there was insufficient data provided in the remaining five studies to allow calculating adjusted odds ratios. However, we calculated crude odds ratios (OR) with 95% confidence intervals (95% CI) for each intervention study and present these results using a summary forest plot without pooling.

## Results

**Fig. 1:**
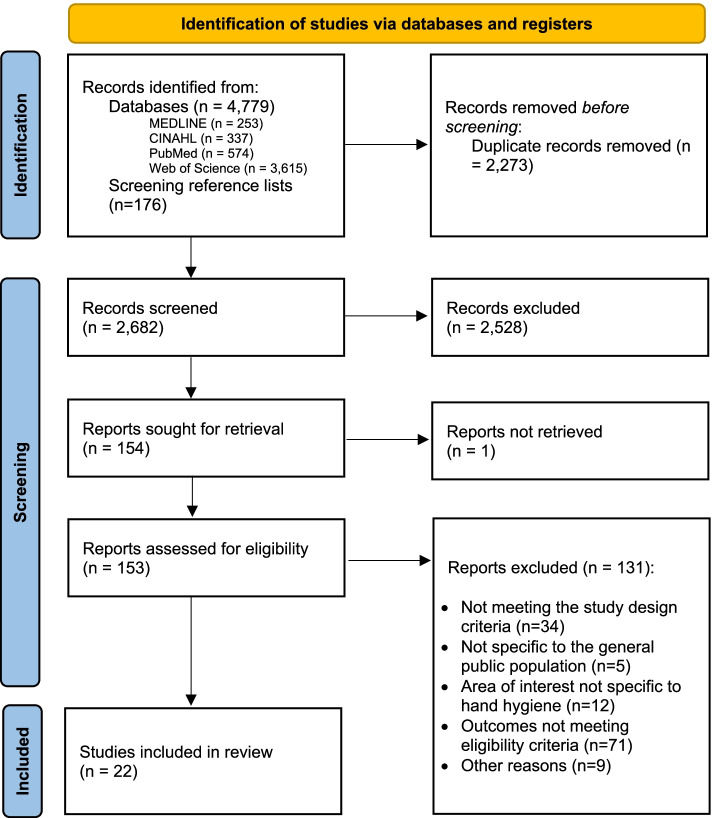
Study selection flow chart

A total of 4955 records were retrieved. After screening of titles and abstracts for eligibility, 153 relevant papers were selected and retrieved for full-text review. Of these, 131 papers were excluded due to the reasons documented in Fig. 1 and additional details presented in Additional file 2.

Of the 22 included studies, six were intervention studies using an RCT or a cluster RCT (cRCT) design and reporting outcomes related to influenza acquisition amongst the general public [19–21] or school children [22–24] (Table 1). With the exception of one study focusing on children [25], all non-intervention studies related to the general public and all focused on risk or protective factors related to acquisition or transmission of influenza (*n* = 5) [25–29], SARS-CoV-1 (*n* = 3) [30–32], or SARS-CoV-2 (*n* = 8) [33–40] (Table 2).

**Table 1:** Intervention studies’ characteristics

Study ID	Country	Aim of the study	Study design	Population	Sample size	Intervention	Comparator	Type of respiratory infection	Relevant outcomes	Findings	Protection effect of HH (effect estimates)
Biswas et al. [24]	Bangladesh	To evaluate the effectiveness of a behaviour change intervention with ABHR and respiratory hygiene messages in school settings to reduce the incidence of influenza virus infections among schoolchildren	cRCT	School children	*N* = 10,855 school children	Provision of ABHR, HH education & respiratory hygiene education	No ABHR provided and no HH & respiratory education provided	Influenza	Influenza acquisition	The incidence of laboratory-confirmed influenza per 1000 student-weeks among children in the intervention schools was 53% lower than in the control schools (IRR: 0.5; 95% CI: 0.3, 0.8; *p* = 0.01).	Significant protective effect (OR: 0.40; 95% CI: 0.22, 0.71)
Cowling et al. [19]	China	To investigate whether HH and use of face masks prevents household transmission of influenza	cRCT	General public	*N* = 259 households	(1) Provision of soap, ABHR & HH education vs. (2) face masks education, provision of surgical masks, soap, ABHR & HH education	Healthy diet and lifestyle education	Influenza	Influenza transmission (secondary attack rates)	Secondary attack ratios (percentage of household members that became infected) did not significantly differ at the household level (24% in the education group, 14% in the HH group, and 18% in the face mask & HH group; *p* = 0.37). However, when intervention was implemented within 36 hours of symptom onset in the index patient, there was a significant difference in the secondary attack ratios between groups (4% in face masks & HH group, 5% in HH group and 12% in education group; *p* = 0.04).	No significant effect (OR: 0.52; 95% CI: 0.27, 1.00)
Larson et al. [20]	USA	To test the effectiveness of three household interventions on incidence & transmission of URI & influenza, knowledge of transmission of URIs, & vaccination rates	cRCT	General public	*N* = 509 primarily Hispanic households	(1) educational materials & ABHR vs. (2) educational materials & ABHR & masks	Educational materials only	Influenza	Influenza acquisition & secondary transmission	No significant difference in influenza cases between the education group and ABHR group (*p* = 0.2) or ABHR & face masks group (*p* = 0.9).	No significant effect (OR: 1.16; 95% CI: 0.67, 2.01)
Ram et al. [21]	Bangladesh	To test the impact of intensive hand washing promotion on household transmission of influenza-like illness and influenza in rural Bangladesh	RCT	General public	*N* = 384 household compounds	Provision of education and skills training, set up of hand washing station and provision of soap & water, provision of reminders (cue cards)	No education or training, no soap or water provided, no hand washing stations, no cue cards	Influenza	Influenza transmission (secondary attack rate ratio)	No significant difference in secondary attack ratio between the intervention arm households (9.6%) and the control arm households (4.0%) of patients with laboratory-confirmed influenza (secondary attack ratio: 2.40; 95% CI: 0.68, 8.47; *p* = 0.17).	No significant effect (OR: 2.52; 95% CI: 1.12, 5.64)
Stebbins et al. [22]	USA	To assess the impact of non-pharmacological interventions on the incidence of laboratory-confirmed influenza infections among elementary school children	cRCT	School children	*N* = 3360 pupils	HH & respiratory hygiene training & ABHR	No ABHR provided and no HH & respiratory training provided	Influenza	Influenza acquisition	No significant effect of the intervention on the number of laboratory-confirmed influenza cases (IRR: 0.81; 95% CI: 0.54, 1.23; *p* = 0.33), but children in intervention schools had significantly fewer influenza A infections in comparison with control schools (IRR: 0.48; 95% CI: 0.26, 0.87; *p* < 0.02).	No significant effect (OR: 0.94; 95% CI: 0.64, 1.39)
Talaat et al. [23]	Egypt	To evaluate the effectiveness of an intensive HH campaign on reducing the incidence of laboratory-confirmed influenza	cRCT	School children	*N* = 44,451 pupils	HH education messages & activities and hand washing twice a day	No HH education provided & no instruction to hand wash twice a day	Influenza	Influenza acquisition	The rate of laboratory-confirmed influenza was higher among pupils who reported their illness in the control schools (35%) than in the intervention schools (18%) (*p* < 0.01).	Significant protective effect (OR: 0.64; 95% CI: 0.51, 0.80)

*ABHR* alcohol-based hand rub, *CI* confidence interval, *cRCT* cluster randomised trial, *HH* hand hygiene, *IRR* incidence rate ratio, *OR* Odds ratio, *p p*-value, *RCT* randomised controlled trial, *URI* upper respiratory infection

**Table 2:** Non-intervention studies’ characteristics

Study ID	Country	Aim of the study	Study design	Population	Sample size	Type of infection	Method of identifying infection	Relevant outcomes	Findings
Abd [33]	Iraq	To identify the risk factors predisposing to SARS-CoV-2 infection	Retrospective cross-sectional survey	General public	*N* = 348 hospitalised COVID-19 cases & 348 hospitalised non-COVID-19 patients	SARS-CoV-2	Confirmed in the hospital (no details provided)	Risk factors for SARS-CoV-2 acquisition	Amongst confirmed COVID-19 cases, 66 (18.96%) reported washing their hands “healthily”, whereas 148 (42.52%) reported doing so sometimes, compared to 94 (27.01%) and 134 (38.50%) non-COVID-19 patients, respectively. There was a statistically significant association between COVID-19 infection and hand washing (*p* < 0.001).
Badri et al. [34]	USA	To identify behaviours and evaluate trends in COVID-19-mitigating practices in a predominantly Black and Hispanic population, to identify differences in practices by self-reported ethnicity, and to evaluate whether federal emergency financial assistance was associated with SARS-CoV-2 acquisition	Retrospective cross-sectional survey	General public	*N* = 209 individuals who tested positive for SARS-CoV-2 & 105 who tested negative	SARS-CoV-2	Laboratory confirmed	Risk factors for SARS-CoV-2 acquisition	Frequent use of ABHR was associated with lower odds of infection (aOR: 0.26; 95% CI: 0.13, 0.52), but frequent hand washing showed no significant effect (aOR: 0.55; 95% CI: 0.21, 1.44).
Castilla et al. [26]	Spain	To evaluate risk factors and measures to prevent influenza infection in the community	Retrospective case-control study	General public	*N* = 481 influenza outpatients & 481 controls	Influenza	Laboratory confirmed	Risk factors for influenza acquisition	The frequency of hand washing 5–10 times (aOR: 0.87; 95% CI: 0.54, 1.39; *p* = 0.56), and > 10 times (aOR: 0.98; 95% CI: 0.59, 1.64; *p* = 0.94), the use of ABHR (aOR: 1.36; 95% CI: 0.85, 2.19; *p* = 0.2) and hand washing after touching contaminated surfaces (aOR: 0.70; 95% CI: 0.44, 1.11; *p* = 0.13) had no significant protective effect.
Doshi et al. [28]	Bangladesh	To identify household-level factors associated with influenza among young children in a crowded community in Dhaka	Prospective case-control study	Pre-school children	*N* = 145 households of influenza paediatric cases & 341 control households	Influenza	Laboratory confirmed	Risk factors for influenza acquisition	The mean hand washing frequency during a 5-hour observation period was similar between case (0.64 events) and control (0.63 events) households (*p* = 0.87). Mean daily soap consumption per capita was 2.92 and 2.93 in the case and control households, respectively (*p* = 0.92). Almost all case (97%) and control (99%) households had water present at the primary hand washing location, but 22% of case households and 29% of control households had soap present before it was provided for soap consumption measurement (*p* = 0.08).
Doung-ngern et al. [35]	Thailand	To evaluate the effectiveness of personal protective measures against SARS-CoV-2 infection in public	Retrospective case-control study	General public	*N* = 211 cases & 839 controls	SARS-CoV-2	Laboratory confirmed	Risk factors for SARS-CoV-2 transmission (secondary attack rates)	A significant, negative association was found between risk for SARS-CoV-2 infection and washing hands “often” (aOR: 0.33; 95% CI: 0.13, 0.87) or “sometimes” (aOR: 0.34; 95% CI: 0.14, 0.81); *p* = 0.045.
Godoy et al. [27]	Spain	To investigate the effectiveness of non-pharmacological interventions in preventing cases of influenza requiring hospitalization	Retrospective case-control study	General public	*N* = 813 hospitalized influenza cases & *N* = 2274 controls	Influenza	Laboratory confirmed	Protective factors against influenza acquisition	The frequency of hand washing 5–10 times (aOR: 0.65; 95% CI: 0.52, 0.84; *p* = 0.001) and > 10 times (aOR: 0.59; 95% CI: 0.44, 0.79, *p* < 0.001) and hand washing after contact with contaminated surfaces (aOR: 0.65; 95% CI: 0.50, 0.84; *p* = 0.001) were protective factors and were dose-responsive (*p* < 0.001). ABHR showed no significant protective effect (aOR: 0.82; 95% CI: 0.65, 1.02; *p* = 0.08).
Karout et al. [36]	USA	To determine the prevalence, level of COVID-19 risk perception attitude and preventive behaviour implemented by the Latino population in the USA	Prospective cross-sectional survey	General public	*N* = 410 asymptomatic Latino adults	SARS-CoV-2	Laboratory-confirmed	Preventive factors associated with SARS-CoV-2 acquisition	Participants who tested positive (*n* = 76; 18.5%) were significantly less likely to use ABHR and wash hands compared with participants who tested negative (*p* < 0.001).
Lau et al. [30]	China	To delineate the distribution of different sources of SARS transmission, identify the undefined source group and to identify relevant risk and protective factors associated with contracting SARS	Retrospective case-control study	General public	*N* = 330 suspected SARS cases with undefined infection sources & 660 controls	SARS-CoV-1	SARS case definition criteria	Risk and preventive factors associated with SARS-CoV-1 acquisition	Frequent hand washing (more than 10 times a day) was a significant protective factor (OR: 0.58; 95% CI: 0.38, 0.87; *p* = 0.008).
Lio et al. [37]	China	To determine the risk and protective factors for COVID-19 infection at the individual level, with a specific emphasis on personal behaviours such as mask use, the number of gatherings, and HH practices	Retrospective case-control study	General public	*N* = 24 hospitalised COVID-19 patients & 1113 control participants who completed quarantine after travelling from COVID-19 high-risk foreign country	SARS-CoV-2	Laboratory-confirmed	Risk and preventive factors associated with SARS-CoV-2 acquisition	Compared to the non-infected individuals, those with SARS-CoV-2 infection were significantly less likely to wash hands after contact with individuals who had respiratory symptoms (50% vs. 95.3%; *p* = 0.005), but not after contact with suspected or confirmed COVID-19 patients (50% vs. 95.2%; *p* = 0.057). Hand washing after outdoor activity (aOR: 0.021; 95% CI: 0.003, 0.134; *p* < 0.005), hand washing before touching the mouth and nose area (aOR: 0.303; 95% CI: 0.114, 0.808; *p* < 0.05) were found to be independent factors for COVID-19 infection. Amongst infected individuals, 16.7% reported always washing hands for over 20 seconds each time, compared with 31.9% in the non-infected group (*p* = 0.125).
Liu et al. [38]	USA	To understand the risk of SARS-CoV-2 transmission from a paediatric primary index case to household contacts living in Los Angeles County	Prospective cohort study	Children	*N* = 15 paediatric index cases & 50 household contacts	SARS-Cov-2	Laboratory-confirmed	Risk factors for SARS-CoV-2 transmission (secondary attack rates)	Overall secondary attack rates were 34% (95% CI: 22, 48%). Transmission was significantly lower in households with increased hand washing or ABHR use compared with those who did not report increased hand washing or ABHR use (19%; 95% CI: 9, 36) vs. 58%; 95% CI: 36, 77; *p* = 0.01).
Speaker et al. [39]	USA	To compare the social behaviours of individuals who were tested positive for COVID-19 relative to non-infected individuals	Retrospective case-control study	General public	*N* = 113 COVID-19 cases & 226 controls	SARS-Cov-2	Laboratory confirmed	Risk factors for SARS-CoV-2 acquisition	67% of cases and 63% of controls reported always washing hands or using ABHR after possible exposures (*p* = 0.24). 75% of cases and 74% of controls reported always washing hands for at least 20 seconds (*p* = 0.60).
Wilson-Clark et al. [31]	Canada	To determine characteristics associated with the transmission of SARS within households	Retrospective cross-sectional survey	General public	*N* = 74 SARS-affected households	SARS-Cov-1	SARS case definition criteria	Risk factors for household transmission of SARS-CoV-1	Failure to wash hands after caring for ill person (RR: 3.46; 95% CI: 1.10, 10.92) and not always washing hands after changing a diaper (RR: 3.94; 95% CI: 1.28, 12.10) were associated with an increased risk of transmission.
Torner et al. [25]	Spain	To investigate the effectiveness of non-pharmaceutical interventions in preventing cases of influenza in children in the community setting in 2009 pandemic and 2010–2011 post pandemic/seasonal epidemic	Retrospective case-control study	Children	*N* = 239 confirmed paediatric influenza outpatients & 239 controls	Influenza	Laboratory-confirmed	Risk factors for influenza acquisition	Hand washing more than 5 times per day (aOR: 0.62; 95% CI: 0.39, 0.99; *p* = 0.04) was the only statistically significant protective factor. For 5–17 age group, there was a negative association for influenza infection for both washing more than 5 times per day (aOR: 0.47; 95% CI: 0.22, 0.99; *p* = 0.04) and hand washing after touching contaminated surfaces (aOR: 0.19; 95% CI: 0.04, 0.86; *p* = 0.03). For the 0–4 years group there was no significant association for washing hands more than 5 times per day (aOR: 0.91; 95% CI: 0.46, 1.78; *p* = 0.77) nor for hand washing after touching contaminated surfaces (aOR: 1.06; 95% CI: 0.44, 2.56; *p* = 0.77).
Wu et al. [32]	China	To compare exposures of unlinked, probable SARS patients with community-based matched controls	Retrospective case-control study	General public	*N* = 94 probable SARS patients & *N* = 281 controls	SARS-Cov-1	Laboratory confirmed	Risk and preventive factors associated with SARS-CoV-1 acquisition	Always washing hands when returning home was a protective factor (OR: 0.3; 95% CI: 0.2, 0.7; *p* = 0.003). No significant association was found for always washing hands before eating (OR: 0.6; 95% CI: 0.3, 1.1; *p* = 0.11) or after using restrooms (OR: 0.5; 95% CI: 0.2, 1.2; *p* = 0.10).
Xie et al. [40]	China	To quantify exposure patterns, transmission characteristics, and the clinical spectrum of SARS-CoV-2 infection	Retrospective cohort study	General public	*N* = 20 index patients hospitalized with severe COVID-19 & 79 of their household contacts	SARS-Cov-2	Laboratory confirmed	Risk factors for SARS-CoV-2 transmission	Hand washing ≥5 times/day was associated with reduced infection risk (52.8% vs.76.9%, *p* = 0.04).
Zhang et al. [29]	China	To assess risk factors associated with household transmission of pandemic H1N1 from self-quarantined patients in Beijing	Retrospective case-control study	General public	*N* = 54 case households & 108 control households	Influenza	Laboratory confirmed	Risk factors for influenza transmission	Hand washing ≥3/day was related to the household transmission of pandemic H1N1 from self-quarantined patients (OR: 0.71; 95% CI: 0.48, 0.94; *p* = 0.05).

*ABHR* alcohol-based hand rub, *aOR* adjusted odds ratio, *CI* confidence intervals, *H1N1* Hemagglutinin Type 1 and Neuraminidase Type 1 (swine flu strain), *p* p-value, *RR* relative risk, *SARS-CoV-1* severe acute respiratory syndrome coronavirus 1, *SARS-CoV-2* severe acute respiratory syndrome coronavirus 2

Studies were categorised according to World Bank definitions [41]. Ten were conducted in high-income countries, including Spain (*n* = 3) [25–27], USA (*n* = 6) [20, 22, 34, 36, 38, 39] and Canada (*n* = 1) [31]. The remaining 12 studies were conducted in upper middle-income countries including, China (*n* = 6) [19, 29, 30, 32, 37, 40], Iraq (*n* = 1) [33] and Thailand (*n* = 1) [35], and lower middle-income countries, Bangladesh (*n* = 3) [21, 24, 28] and Egypt (*n* = 1) [23].

### Intervention studies

#### Households

Two cRCTs [19, 20] and one RCT [21] focused on households. The studies were conducted in New York in 2006–08 amongst 509, mainly Latino households [20], in Hong Kong during 2008 amongst 259 households of patients, who presented with symptoms of acute respiratory illness [19] and in rural area in Bangladesh in 2009–10 amongst 384 household compounds of index case-patients. In all studies, households or compounds were randomised into study arms. In both Larson et al. [20] and Cowling et al. [19], there were three study arms, including education only, education and ABHR and education with ABHR and mask wearing by caretaker and person showing symptoms of influenza. In Ram et al. [21] the intervention consisting of education, set up of hand washing stations, provision of soap and water, and HH cue cards, was compared against the control arm in which no intervention was applied. The outcome measure in all three studies were laboratory-confirmed influenza incidence [19] and/or influenza transmission (i.e. secondary attack rate) within the household [19–21].

Larson et al. [20] found the group, which received ABHR and education regarding ABHR use and the prevention of respiratory infections, included significantly more household members without any symptoms (57.6%) as compared with the education group (49.4%) and the education, ABHR and face mask group (38.7%) (*p* < 0.01). However, no significant difference in influenza acquisition rates was found between the education group and education and ABHR (*p* = 0.2) or education, ABHR and face mask group (*p* = 0.9). Likewise, Ram et al. [21] observed no significant difference in secondary attack ratio amongst the susceptible members of the households of laboratory-confirmed index patients in the intervention compounds (9.6%) and the control compounds (4.0%) (2.40, 95% CI: 0.68, 8.47; *p* = 0.17). However, as noted it is not clear from the studies when HH was performed.

Cowling et al. [19] demonstrated some effect of an HH intervention, consisting of soap and ABHR provision, demonstration of correct HH technique and education about the importance of HH for preventing influenza transmission. Household-level secondary attack ratios were 14% in the HH group in comparison with 24% in the education-only group, and 18% in the HH and face masks group. However, this difference was not significant (*p* = 0.37). Yet, a significant difference in the secondary attack ratios was found between the groups if the intervention was implemented within 36 hours of symptom onset in the index patient (4% in face masks & HH group, 5% in an HH group and 12% in education only group; *p* = 0.04), suggesting a benefit in early implementation of a combination of HH and face masks. But the relative contributions of the interventions were not shown; thus, the individual effect of each of these two interventions is not known and it remains uncertain how these two interventions effected the outcomes.

#### School children

Three cRCTs focusing on elementary school children, including 44,451 pupils from 60 schools in Cairo in 2008 [23], 3360 pupils from 10 schools in Pittsburgh during the 2007–2008 influenza season [22] and 10,855 pupils from 24 schools in Dhaka in 2015 influenza season [24]. In each study, participating schools were randomised to an educational intervention or control group which received no intervention. Interventions consisted of HH education through entertainment activities, booklets and posters and washing hands at least twice a day [23], HH and respiratory hygiene training, presentation of correct HH and teaching children to use it at least four times a day [22], or provision of ABHR in classrooms and outside the toilets, provision of training to teachers, and incorporating HH and respiratory hygiene education into curriculum [24]. Furthermore, in Stebbins et al. [22] and Biswas et al. [24], children were taught to use ABHR at specific times, i.e., upon arrival to school or entering the classroom, when leaving school, before and/or after lunch [23, 24], after sneezing, coughing, or blowing their nose and after using the toilet [24].

Talaat et al. [23] found the rate of laboratory-confirmed influenza was higher among pupils who reported their illness in the control schools (35%) than in the intervention schools (18%; *p* < 0.01). A significant effect of the intervention was also observed by Biswas et al. [24] with the incidence of laboratory-confirmed influenza per 1000 student-weeks among children in the intervention schools found to be 53% lower than in the control schools (incidence rate ratio [IRR]: 0.5; 95% CI: 0.3, 0.8; *p* = 0.01). Stebbins et al. [22] reported significantly fewer influenza A infections in the intervention schools, in comparison with control schools (IRR: 0.48; 95% CI: 0.26, 0.87; *p* < 0.02); however, observed no significant effect of the intervention when the total number of laboratory-confirmed influenza cases was considered (IRR: 0.81; 95% CI: 0.54, 1.23; *p* = 0.33).

#### Effect estimates of the intervention studies

**Fig. 2:**
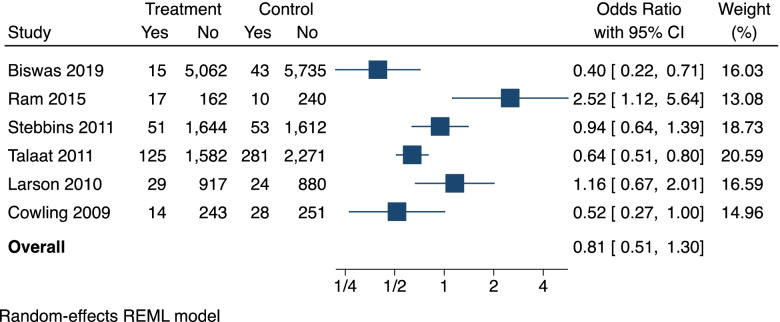
Forest plot showing the individual effects of hand hygiene interventions on laboratory confirmed influenza

Figure 2 displays the effect estimates (crude odds ratios) of the six interventions studies. Four of the studies [19, 22–24] had odd ratios of less than one, indicative of a protective effect of the HH intervention against influenza; however, this effect was only statistically significant in the two largest studies by Talaat et al. [23] (OR: 0.64; 95% CI: 0.51, 0.80) and Biswas et al. [24] (OR: 0.40; 95% CI: 0.22, 0.71). The intervention in Ram et al. [21] was associated with a significant increase in influenza (OR: 2.52; 95% CI: 1.12, 5.64) and Larson et al. [20], was associated with a non-significant increase in influenza (OR: 1.16; 95% CI: 0.67, 2.01).

### Non-intervention studies

As shown in Table 2, most of the studies used case-control design [25–30, 32, 35, 37, 39] with four studies being cross-sectional surveys [31, 33, 34, 36], and two using a cohort design [38, 40]. The studies focused on hand washing or ABHR use, frequency of HH or when it was performed as a risk or protective factors for the transmission or acquisition of influenza [25–29], SARS-CoV-1 [30–32] or SARS-CoV-2 [33–40].

#### Influenza

Of the five studies that investigated the protective or risk factors for influenza acquisition [25–28] or transmission [29] all used case-control design. With the exception of Torner et al. [25] who focused on children, all studies concerned general public population. Furthermore, in all five studies, the presence of influenza infection was laboratory-confirmed.

Two studies [28, 29] concerned the frequency of hand washing with soap and water exclusively. The study by Zhang et al. [29], conducted between August and September 2009 in Beijing, involved 162 households of self-quarantined pandemic H1N1 influenza index patient and 108 control households found that washing hands at least three times a day was a protective factor against influenza transmission (OR: 0.71; 95% CI: 0.48, 0.94; *p* = 0.05). Doshi et al. [28] on the other hand, conducted 5-hour direct observations of HH practices in 145 households of laboratory-confirmed influenza A and B paediatric cases 4–6 weeks after the diagnosis, and 341 control households in Dhaka between March 2009 and March 2010. No significant difference was found in HH frequency between case (0.64 events) and control (0.63 events) households (*p* = 0.87).

The remaining three Spanish studies were multisite and all investigated the protective effect of hand washing frequency (more than 5 times a day [25] or 5–10 times a day and more than 10 times a day [26, 27]), washing hands after touching contaminated surfaces, and the use of ABHR on influenza acquisition. Castilla et al. [26] and Godoy et al. [27] were hospital studies of community HH behaviours prior to hospital attendance, conducted between 2009 and 2010 in 36 Spanish hospitals [26, 27]. However, in Castilla et al. [26] the sample consisted of 481 confirmed influenza adult outpatients and 481 controls (ambulatory primary health care patients who consulted for reasons other than influenza or respiratory symptoms), matched for age, date of consultation and province of residence. In Godoy et al. [27], 813 hospitalized, confirmed influenza cases were matched with 2274 controls (patients with unplanned hospital admissions and patients attending primary health care for reasons other than influenza-like illness), using the same criteria as those reported in Castilla et al. [26]. Furthermore, Castilla et al. [26] reported their investigation as being part of a larger study evaluating the effectiveness of various measures in preventing influenza. Although not explicitly stated, Godoy et al. [27] appears to be a sub-analysis of the same larger study. The third Spanish study [25] focused specifically on the H1N1 influenza amongst children aged 6 months – 17 years, with the sample size of 239 paediatric influenza outpatients and 239 controls during the 2009–10 and 2010–11 seasons.

Despite methodological similarities, the findings of the three Spanish studies were inconsistent. Castilla et al. [26] reported that neither the frequency of hand washing 5–10 times (adjusted odds ratio [aOR]: 0.87; 95% CI: 0.54, 1.39; *p* = 0.56), nor more than 10 times a day (aOR: 0.98; 95% CI: 0.59, 1.64; *p* = 0.94), nor ABHR use (aOR: 1.36; 95% CI: 0.85, 2.19; *p* = 0.2) nor habitual hand washing after touching contaminated surfaces (aOR: 0.70; 95% CI: 0.44, 1.11; *p* = 0.13) had a significant protective effect. Conversely, Godoy et al. [27] found that washing hands 5–10 times a day (aOR: 0.65; 95% CI: 0.52, 0.84; *p* = 0.001) or more than 10 times a day (aOR: 0.59; 95% CI: 0.44, 0.79; *p* < 0.001) and washing hands after contact with contaminated surfaces (aOR: 0.65; 95% CI: 0.50, 0.84; *p* = 0.001) all had a significant protective effect against influenza hospitalisation and were dose-responsive (*p* < 0.001). However, like Castilla et al. [26], Godoy et al. [27] reported that ABHR showed no significant benefits (aOR: 0.82; 95% CI: 0.65, 1.02; *p* = 0.08), possibly because of insufficient instruction provided to the participants. Finally, Torner et al. [25] reported that hand washing more than 5 times per day was a protective factor (aOR: 0.62; 95% CI: 0.39, 0.99; *p* = 0.04) against influenza acquisition. However, hand washing after touching contaminated surfaces was found to be a protective factor amongst the 5–17 age group (aOR: 0.19; 95% CI: 0.04, 0.86; *p* = 0.03), but not for the 0–4 years group (aOR: 1.06; 95% CI: 0.44, 2.56; *p* = 0.77). Yet, like in Castilla et al. [26] and Godoy et al. [27], the use of ABHR had no significant protective effect (aOR: 1.54; 95% CI: 0.8, 2.66; *p* = 0.13) [25].

#### SARS

Three studies focused on SARS [30–32]. These included two retrospective matched case-controlled studies conducted in Hong Kong [30] and Beijing [32] in 2003, and a cross-sectional survey conducted between May–October 2003 in Toronto [31]. All three studies focused on the general public population and all used SARS case definition criteria to identify cases.

Of the three studies, one [30] looked at the frequency of hand washing. The study involved conducting telephone interviews with 330 undefined source SARS cases and 660 controls, matched for age and sex, and demonstrated that washing hands more than 10 times a day was a significant protective factor (OR: 0.58; 95% CI: 0.38, 0.87; *p* = 0.008) [30].

The remaining two studies [31, 32], focused on when hand washing should be performed. Wu et al. [32], used standardised questionnaires, delivered in person or by telephone interviews, to compare exposures of 94 unlinked, SARS patients with those of 281 community-based controls matched for age group, region, and sex, while Wilson-Clark et al. [31] collected data, using structured interviews, from 74 SARS-affected households, identified using SARS case definition and representing a third of all households impacted in the region.

Wu et al. [32] reported that always washing hands when returning home had a protective effect (OR: 0.3; 95% CI: 0.2, 0.7; *p* = 0.003). However, no significant association was found for always washing hands before eating (OR: 0.6; 95% CI: 0.3, 1.1; *p* = 0.11) or after using restrooms (OR: 0.5; 95% CI: 0.2, 1.2; *p* = 0.10) [32]. Wilson-Clark et al. [31] reported that failure to wash hands after caring for an ill person (relative risk [RR]: 3.46; 95% CI: 1.10, 10.92) and not always washing hands after changing a diaper (RR: 3.94; 95% CI: 1.28, 12.10) were associated with an increased transmission risk [31].

#### COVID-19

Eight studies, including three case-control studies [35, 37, 39], three cross-sectional surveys [33, 34, 36] and two cohort studies [38, 40] investigated the risk or protective factors for SARS-CoV-2 acquisition [33, 34, 36, 37, 39] or transmission [35, 38, 40] in the general public population. With the exception of one study [38], conducted between December 2020 – February 2021, all were conducted in 2020, with the length of study varying from 9 days [33] to 2 months [34]. Furthermore, in all studies SARS-CoV-2 transmission or acquisition was confirmed by laboratory tests, with the exception of Abd et al. [33], in which COVID-19 infections were confirmed in the hospital, but no details were provided.

Five studies [34–36, 38, 40], investigated risk or protective factors related to the frequency of HH. Karout et al. [36] and Liu et al. [38] both investigated the effect of frequent HH, performed either by the means of washing hands with soap and water or handrubbing with ABHR, amongst 410 asymptomatic Latino adults in Maryland [36] or 15 paediatric index cases and their 50 household contacts in Los Angeles [38]. Karout et al. [36] found a protective effect of frequent HH against SARS-CoV-2 acquisition (*p* < 0.001), while Liu et al. [38] found that secondary attack rates were significantly lower in households with increased hand washing or use of ABHR compared to households not reporting increased HH (19% [95% CI: 9, 36] vs. 58% [95% CI: 36, 77], *p* = 0.01). Furthermore, Doung-ngern et al. [35] studied 211 cases and 839 controls in Thailand and demonstrated that frequent hand washing with soap and water was a protective factors against SARS-CoV-2 transmission (*p* = 0.045). However, another study [34] of 209 positive cases and 105 controls in Chicago showed that while frequent use of ABHR was associated with significantly lower odds of testing positive for SARS-CoV-2 (aOR: 0.26; 95% CI: 0.13, 0.52), frequent hand washing was not (aOR: 0.55; 95% CI: 0.21, 1.44). Only one study [40] involving 20 index patients hospitalized with severe COVID-19 in Beijing and 79 of their household contacts investigated a specific frequency of hand washing and showed that hand washing at least 5 times per day was associated with reduced risk of transmission (52.8% vs.76.9%; *p* = 0.04).

One study [33], conducted amongst 348 hospitalised COVID-19 cases and 348 patients hospitalised for other reasons in Al-Nasiriya city in Iraq, investigated “healthy hand washing”; however, provided no details on what this involved. Nevertheless, “healthy hand washing” was significantly associated with lower risk of infection (*p* < 0.001).

Two studies [37, 39] focused on both, the duration of hand washing (for at least 20 seconds) and hand washing or ABHR use at specific times, including after contact with high-risk [39] or symptomatic [37] individuals, washing hands when hands are visibly dirty, before eating, before or after handling food, after using toilet, after outdoor activity, before or after attending to a child or sick person, after sneezing or coughing, after handling pets and before touching the mouth or nose area [37]. The studies were conducted in Ohio and Florida, with the involvement of 113 COVID-19 cases and 226 controls [39] and in Macao, China amongst 24 patients hospitalised for COVID-19 and 1113 control participants who completed quarantine after travelling from a COVID-19 high-risk foreign country [37].

Lio et al. [37] reported that washing hands after outdoor activity (aOR: 0.021; 95% CI: 0.003, 0.134; *p* < 0.005) and before touching the mouth and nose area (aOR: 0.303; 95% CI: 0.114, 0.808; *p* < 0.05) were found to be independent protective factors against SARS-CoV-2 acquisition. Interestingly, Lio et al. [37], also found that compared to control participants, infected individuals were significantly less likely to report washing hands after contact with someone who had respiratory symptoms (50% vs. 95.3%; *p* = 0.005), but not after contact with suspected or confirmed COVID-19 patients (50% vs. 95.2%; *p* = 0.057), while Speaker et al. [39] found no significant effect of washing hands or using ABHR after possible exposures (*p* = 0.24). In addition, neither Speaker et al. [39] nor Lio et al. [37] found a significant association between always washing hands for at least 20 seconds and SARS-CoV-2 acquisition (*p* = 0.125 [37]; *p* = 0.60 [39]).

### Quality assessment

With the exception of one RCT [21], all intervention studies were cRCTs. With the exception of Talaat et al. [23], which was assessed as unclear risk of bias due to insufficient reporting, the overall risk of bias was high with at least one item assessed as high risk; (Additional file 3: Table A).

All non-intervention studies were observational with one prospective [36] and three retrospective cross-sectional surveys [31, 33, 34], one prospective [28] and nine retrospective case-control studies [25–27, 29, 30, 32, 35, 37, 39], and one prospective [38] and one retrospective cohort study [40]. Apart from one cross-sectional study [31], all non-intervention studies were assessed as unclear [25, 29, 30, 34, 40] or high [26–28, 32, 33, 35–39] risk of bias. Details are presented in Additional file 3: Table B-D.

## Discussion

This review evaluated available literature on the effectiveness of HH as an intervention for prevention of community transmission or acquisition of respiratory viruses that have caused epidemics or pandemics and whether HH is a protective factor against acquisition or transmission of such infections in the community. There is limited evidence suggesting that encouraging HH could be beneficial for prevention of SARS-CoV-1, SARS-CoV-2, and influenza viruses in the community and showed that HH interventions could be effective in preventing influenza in school children.

The review is unique in its specific focus on the role of HH in the transmission or acquisition of novel coronaviruses or influenza viruses, which are of importance because they cause epidemics and pandemics. While other recent systematic reviews [42–44] focused on the effectiveness of HH in preventing respiratory infections, these had a broader scope of public health measures, such as the combination of face masks, HH or social distancing [43], focused on HH promotion programmes [44], focused exclusively on RCTs [43, 44] or on evidence from low- to middle-income countries [42].

Our analysis of effect estimates of the intervention studies showed, that educational interventions paired with washing hands at least twice daily [23] or provision of ABHR and instruction to use it at specific moments [24] had significant protective effect against influenza acquisition in school children. Talaat et al. [23] and Biswas et al. [24] were the two largest studies; and while the risk of bias was assessed as high for Biswas et al. [24], Talaat et al. [23] was the only intervention study for which the overall risk of bias was unclear. Nevertheless, this is likely to be the result of the limited reporting, rather than methodological weaknesses. Thus, the intervention evaluated by Talaat et al. [23] in Egypt is likely to be beneficial for preventing influenza when implemented in other, similar school settings.

Although non-significant, two intervention studies [19, 20] looked at the combined effect of face masks and HH, the contribution of HH to the collective effect of these measures remains uncertain. It is possible that face mask use and HH are connected behaviours, because HH is embedded within the correct mask use [45, 46]. Furthermore, increased motivation to exert self-protective behaviours as a result of perceived threat [47] is likely to result in an increased adherence to guidelines in general. Thus, further research should consider the individual contributions of different intervention components.

Evidence derived from the non-intervention studies indicate that encouraging HH could be beneficial for preventing acquisition or transmission of SARS-CoV-1, SARS-CoV-2 and influenza viruses in the general public population. Overall, 13/16 studies showed significant effect. HH was either promoted in relation to how often or in what circumstances it was performed with most evidence being for the frequency.

Of the 11 studies that investigated for the protective effect of frequent or increased HH, nine [25, 27, 29, 30, 34–36, 38, 40] demonstrated a significant effect; however, these studies focused on different types of infections and there is no doubt that many feature a number of limitations in understanding in detail behaviours around HH. Amongst the SARS-CoV-2 studies that investigated the effect of frequent or increased HH, all five [34–36, 38, 40] demonstrated a significant protective effect. However, only one [40] of these studies investigated a specific frequency of washing hands at least five times a day, while the remaining four studies did not provide information on how many times a day HH should be performed for the protective effect. For influenza and SARS, evidence was less consistent. For influenza only three [25, 27, 29] out of five studies demonstrated a significant protective effect of frequent HH. While three studies investigated specific HH frequency rates, the frequencies differed across these studies. Finally, of the studies focusing on SARS-CoV-1, only one [30] investigated for the protective effect of specific HH frequency and showed that washing hands at least 10 times a day was protective against SARS-CoV-1 acquisition. Single pieces of evidence for SARS-Cov-2 and SARS-Cov-1, derived from relatively small study is insufficient to make recommendations regarding how often HH should be performed to prevent SARS and COVID-19 infections in the community.

This review has not found consistent evidence as to when the publics’ hands should be cleaned. While factors related to such circumstances were investigated in seven [25–27, 31, 32, 37, 39] of the non-intervention studies, only five [25, 27, 31, 32, 37] had a significant effect. These were context-specific, and findings were inconsistent. The lack of consistent evidence of when hands should be cleaned is concerning given that expert-informed HH guidelines for healthcare staff [10, 11], indicate it is essential to perform HH at specific, defined times, or ‘moments’ associated with increased risk of hand contamination, rather than simply recommending frequent HH.

Another aspect of HH investigated in the non-intervention studies was how long HH should be performed for. However, of all studies included in our review, only two [37, 39] investigated for the protective effect of washing hands for at least 20 seconds, and both reported a lack of significant effect.

Finally, the choice of HH products was also considered. In our review, only four [25–27, 34] of the non-intervention studies investigated the effects of using ABHR rather than hand washing with soap. Two [25, 34] of these reported a significant protective effect of use of ABHR resulting in no body of evidence as one was protective against SARS-CoV-2 [34] and the other against influenza H1N1 [25] acquisition.

Thus, for the non-intervention studies, there is only a body of evidence supporting the promotion of HH to prevent the acquisition or transmission of SARS-CoV-2 [33–38, 40]. However, this evidence supports frequent or increased HH but does not specify the frequency in which, or timings when HH should be performed. Moreover, confounding factors could have impacted the validity and reliability of these findings. These included participants inability to recall how many times a day or when they cleaned their hands in studies using retrospective self-report for measurement of HH behaviour [33–35, 37, 40], potential lack of technique when participants performed self-swabbing for testing purposes [36, 38] and participants tendency to inflate their HH behaviour and give the expected responses when interviewed by researchers [34–36]. Relatively small sample sizes across the body of evidence but particular for Badri et al. [34] and Karout et al. [36] and descriptive analysis of the data [36] further confounded the findings of the studies. However, given that 9 out of 11 studies investigating frequency of HH as a protective measure showed significant effect, and 13/16 studies showed significant protective effect of some aspect of HH against transmission or acquisition of these respiratory viral infections in the community, it is recommended that HH is promoted during epidemics or pandemic. However, further research is warranted should the opportunity arise to explore how frequent HH should be performed, and in what specific circumstances.

To be impactful, HH recommendations should be consistent and simple enough for the public to put into practice. The public need to be told specifically when and how to clean their hands during pandemics or epidemics [48]. Communication campaigns may need to be tailored for different contexts and community groups [49, 50]. Such patterning of intervention effectiveness across different populations groups has been observed by others. For example, in relation to improving the public’s antimicrobial resistance awareness and behaviours [51] or using apps for improving lifestyle behaviours [52]. In addition, a recent integrative review [53] found that engagement with protective behaviours within the community, including HH, can be influenced by demographic, social and psychological factors. Thus, it is important to consider contextual and individual factors when planning future interventions. Furthermore, improving knowledge alone is often insufficient to achieve desirable behaviour change; thus, campaigns should aim to motivate individuals to take action by making the intended behaviour change appealing [50]. This is consistent with a multimodal approach to HH in healthcare improvement tested and implemented by WHO for over a decade [54, 55].

Epidemics and pandemics provide opportunities to encourage protective behaviours in the communities [56], possibly because the perceived threat is likely to motivate individuals to change their habits, as suggested by the Health Belief Model [47]. As demonstrated by a Japanese survey conducted amongst 2149 members of the public, the mean self-reported HH frequency during COVID-19 pandemic was 10.2 times per day [57]; thus, a frequency exceeding that found to be significantly protective in five of the studies in our review [25, 27, 29, 30, 40]. However, without continuous reinforcement these behaviours are likely to diminish over time [56], emphasising the need for continuous reinforcement.

### Limitations

None of the six intervention studies included in our review focused on COVID-19. Therefore, while these studies provide limited evidence for the effectiveness of the HH interventions for prevention of influenza infections, the effectiveness of such interventions in the current context of COVID-19 pandemic remains unknown. Nevertheless, the review identified eight non-intervention studies focusing on SARS-CoV-2 [33–40], and another eight non-intervention studies that focused on other respiratory virus infections that have caused epidemics or pandemics, including SARS and influenza.

### Recommendations

The current evidence is limited by the amount of intervention studies, their focus on influenza prevention, and methodological quality; thus, further intervention research using robust study designs and focusing specifically on SARS-CoV-2 virus is required. To develop clear and simple guidance for the public, further work should focus on identifying the specific times when HH should be performed in different communities and situations. In the meantime, current guidelines should be followed and should be based on evidence summarised here [12, 13, 49, 58, 59]. Resources to support frequent hand washing, if hand washing facilities are available, or alternatively ABHR, should be provided in schools, workplaces, and public spaces and HH should continue to be promoted. While public communication campaigns might require tailoring for specific sub-populations and context, further studies could inform how they can be constructed to convey consistent and simple messages to motivate desired behavioural changes.

## Conclusions

To our knowledge this is the first systematic review focusing specifically on the effectiveness of HH in preventing community transmission or acquisition of novel coronaviruses or influenza viruses that have caused epidemics or pandemics. We have conducted a comprehensive systematic search and review and reported our work in adherence with the PRISMA statement [15] to enhance the rigour. Although it was not appropriate to perform a meta-analysis, we conducted a sub-group quantitative analysis of intervention studies to quantify the protective effect of the HH interventions against influenza transmission or acquisition. Finally, all reviewed RCTs and cRCTs used laboratory testing to confirm respiratory infection, ensuring objectivity of outcome measurement.

Our review summarizes the evidence on the effectiveness of HH against the transmission or acquisition of SARS-CoV-1, SARS-CoV-2 or influenza viruses. While there is weak evidence suggesting that encouraging HH could be beneficial for preventing acquisition or transmission of SARS-CoV-1, SARS-CoV-2 and influenza viruses, these findings mainly derive from non-intervention studies and are limited by methodological quality and heterogeneity of the evidence. Furthermore, the evidence is inconclusive in relation to frequency or exact times for HH and the protective effect of using ABHR. Thus, there is no evidence to suggest changes to current guidelines [49, 58, 59]. Future work is required to outline when and how often HH should be performed in different community settings and to develop innovative, targeted, and effective interventions for promoting good HH habits in communities.

## Supplementary Information

**Additional file 1.** Search strategy applied to MEDLINE database.

**Additional file 2.** Excluded studies with reasons.

**Additional file 3.** Quality Assessment Tables (Table A. Risk of bias of randomised controlled trials; Table B. Quality assessment of case-controlled studies; Table C. Quality assessment of cross-sectional studies; Table D. Quality assessment of cohort studies).

## Data Availability

The datasets used and/or analysed during the current study are available from the corresponding author on reasonable request.

## Abbreviations

ABHR: Alcohol-based hand rub
aOR: Adjusted odds ratio
CI: Confidence interval
COVID-19: Coronavirus disease 2019
cRCT: Cluster randomised controlled trial
H1N1: Hemagglutinin Type 1 and Neuraminidase Type 1
HH: Hand hygiene
IRR: Incidence rate ratio
MERS: Middle East respiratory syndrome
OR: Odds ratios
PRISMA: Preferred Reporting Items for Systematic Reviews and Meta-Analyses
RCT: Randomised controlled trial
RR: Relative risk
SARS: Severe acute respiratory syndrome
WHO: World Health Organization
