# Supplementary material for: The effectiveness of hand hygiene interventions for preventing community transmission or acquisition of novel coronavirus or influenza infections: a systematic review
Source: BMC Public Health. 2022 Jul 2;22:1283. doi: 10.1186/s12889-022-13667-y (PMC9250256; doi:10.1186/s12889-022-13667-y)
Supplement: Supplementary file 1 — Additional file 1. Search strategy applied to MEDLINE database. [file 12889_2022_13667_MOESM1_ESM.docx]

# Additional file 1. Search strategy applied to MEDLINE database

|  | **Free text words** | **Mesh term(s)** |
| --- | --- | --- |
| Population and settings |  | Humans |
|  | Public, population*, communit*, citizen*, resident*, people  Household*, home, homes, hostel*, dormitor*, retirement facilit*, sheltered hous*, sheltered accommodation*, supported living,  family, families, adolescent*, teenager*  shop*, park*, market* public space*, public place*, public area*, public premise*, public venue*, public service*, church*, chapel*, mosque*, synagogue* | Public Health  Community Health Services  Schools, Nursery  Caregivers  Adult  Men  Women  Child  Parents  Fathers  Mothers  Students  Transportation  Toilet Facilities |
| Area of Interest |  | hand hygiene |
| Context | Covid-19, SARS-CoV-2, MERS, respiratory virus* | Severe Acute Respiratory Syndrome  SARS Virus  Middle East Respiratory Syndrome Coronavirus  Coronavirus  Betacoronavirus  Coronavirus Infections |
| Publication date | Restrict to sources published in or after 2002 (as according to CDC, first SARS case was reported in Nov 2002 <https://www.cdc.gov/about/history/sars/timeline.htm>) | |
| Combined search | S1 AND S2 AND S3 AND S4 AND S5 AND S6 AND S7 | |
