# Supplementary material for: The effectiveness of hand hygiene interventions for preventing community transmission or acquisition of novel coronavirus or influenza infections: a systematic review
Source: BMC Public Health. 2022 Jul 2;22:1283. doi: 10.1186/s12889-022-13667-y (PMC9250256; doi:10.1186/s12889-022-13667-y)
Supplement: Supplementary file 2 — Additional file 2. Excluded studies with reasons. [file 12889_2022_13667_MOESM2_ESM.docx]

# Additional files 2. Excluded studies with reasons

**Not meeting the study design criteria (n=34)**

**Non-primary research design (n=5)**

1. Beiu C, Mihai M, Popa L, Cima L, Popescu MN. Frequent Hand Washing for COVID-19 Prevention Can Cause Hand Dermatitis: Management Tips. Cureus. 2020; 12(4).

2. Ramesh N, Siddaiah A, Joseph B. Tackling corona virus disease 2019 (COVID 19) in workplaces. Indian Journal of Occupational and Environmental Medicine. 2020; 24(1):16-18.

3. Savolainen-Kopra C, Haapakoski J, Peltola PA, Ziegler T, Korpela T, Anttila P et al. STOPFLU: is it possible to reduce the number of days off in office work by improved hand-hygiene? Trials. 2010; 11(1):69.

4. World Health Organization Writing Group. Nonpharmaceutical interventions for pandemic influenza, national and community measures. Emerging Infectious Diseases. 2006; 12(1):88-94.

5. Dominguez A, Alonso J, Astray J, Baricot M, Canton R, Castilla J et al. Risk factors of influenza (H1N1) 2009 hospitalization and effectiveness of pharmaceutical and nonpharmaceutical interventions in its prevention: a case-control study. Revista española de salud pública. 2011; 85(1):3-15.

**Modelling study (n=1)**

1. Agossou O, Atchadé MN, Djibril AM. Modeling the effects of preventive measures and vaccination on the COVID-19 spread in Benin Republic with optimal control. Results in Physics. 2021; 31:104969.

**Pilot of already identified study (n=1)**

1. Cowling BJ, Fung RO, Cheng CK, Fang VJ, Chan KH, Seto WH et al. Preliminary findings of a randomized trial of non-pharmaceutical interventions to prevent influenza transmission in households. PloS One. 2008; 3(5):e2101.

**Review studies (n=27)**

1. Jefferson T, Del Mar C, Dooley L, Ferroni E, Al-Ansary LA, Bawazeer GA et al. Physical interventions to interrupt or reduce the spread of respiratory viruses: systematic review. Bmj-British Medical Journal. 2009; 339:B3675-B3675.

2. Jefferson T, Del Mar C, Dooley L, Ferroni E, Al-Ansary LA, Bawazeer GA et al. H1N1 influenza and pandemic flu A special themed issue of the Health Technology Assessment journal series. Health Technology Assessment. 2010; 14(34):355-+.

3. Jefferson T, Del Mar C, Dooley L, Ferroni E, Al-Ansary LA, Bawazeer GA et al. Physical interventions to interrupt or reduce the spread of respiratory viruses. Cochrane Database of Systematic Reviews. 2010(1):6207-6207.

4. Jefferson T, Foxlee R, Del Mar C, Dooley L, Ferroni E, Hewak B et al. Interventions for the interruption or reduction of the spread of respiratory viruses. Cochrane Database of Systematic Reviews. 2007(4):6207-6207.

5. Jefferson T, Foxlee R, Del Mar C, Dooley L, Ferroni E, Hewak B et al. Physical interventions to interrupt or reduce the spread of respiratory viruses: systematic review. BMJ: British Medical Journal (International Edition). 2008; 336(7635):77-80.

6. Aiello AE, Coulborn RM, Aragon TJ, Baker MG, Burrus BB, Cowling BJ et al. Research findings from nonpharmaceutical intervention studies for pandemic influenza and current gaps in the research. American Journal of Infection Control. 2010; 38(4):251-258.

7. Aiello AE, Coulborn RM, Perez V, Larson EL. Effect of hand hygiene on infectious disease risk in the community setting: a meta-analysis. American Journal of Public Health. 2008; 98(8):1372-1381.

8. Aiello AE, Larson EL. What is the evidence for a causal link between hygiene and infections? The Lancet Infectious Diseases. 2002; 2(2):103-110.

9. Benkouiten S, Charrel R, Belhouchat K, Drali T, Salez N, Nougairede A et al. Circulation of respiratory viruses among pilgrims during the 2012 Hajj pilgrimage. Clinical Infectious Diseases. 2013; 57(7):992-1000.

10. bin-Reza F, Chavarrias VL, Nicoll A, Chamberland ME. The use of masks and respirators to prevent transmission of influenza: a systematic review of the scientific evidence. Influenza and Other Respiratory Viruses. 2012; 6(4):257-267.

11. Jefferson T, Del Mar CB, Dooley L, Ferroni E, Al-Ansary LA, Bawazeer GA et al. Physical interventions to interrupt or reduce the spread of respiratory viruses. Cochrane Database of Systematic Reviews. 2011(7):6207-6207.

12. Rabie T, Curtis V. Handwashing and risk of respiratory infections: a quantitative systematic review. Tropical Medicine & International Health. 2006; 11(3):258-267.

13. Warren‐Gash C, Fragaszy E, Hayward AC. Hand hygiene to reduce community transmission of influenza and acute respiratory tract infection: a systematic review. Influenza and Other Respiratory Viruses. 2013; 7(5):738-749.

14. Abdullahi L, Onyango JJ, Mukiira C, Wamicwe J, Githiomi R, Kariuki D et al. Community interventions in Low-And Middle-Income Countries to inform COVID-19 control implementation decisions in Kenya: a rapid systematic review. PloS one. 2020; 15(12):e0242403.

15. Aggarwal N, Dwarakanathan V, Gautam N, Ray A. Facemasks for prevention of viral respiratory infections in community settings: A systematic review and meta-analysis. Indian journal of public health. 2020; 64(6):192.

16. Arefi MF, Poursadeqiyan M. A review of studies on the COVID-19 epidemic crisis disease with a preventive approach. Work. 2020; 66(4):717-729.

17. Chan EYY, Shahzada TS, Sham TST, Dubois C, Huang Z, Liu S et al. Narrative review of non-pharmaceutical behavioural measures for the prevention of COVID-19 (SARS-CoV-2) based on the Health-EDRM framework. British medical bulletin. 2020; 136(1):46.

18. Hoffmann T, Bakhit M, Krzyzaniak N, Del Mar C, Scott AM, Glasziou P. Soap versus sanitiser for preventing the transmission of acute respiratory infections in the community: a systematic review with meta-analysis and dose–response analysis. BMJ open. 2021; 11(8):e046175.

19. Jefferson T, Del Mar CB, Dooley L, Ferroni E, Al-Ansary LA, Bawazeer GA et al. Physical interventions to interrupt or reduce the spread of respiratory viruses. Cochrane Database of Systematic Reviews. 2020(11):Art. No.: CD006207.

20. Medina C, Chavira J, Aburto T, Nieto C, Contreras-Manzano A, Segura L et al. Rapid review: evidence of Covid-19 transmission and similar acute respiratory infections in open public spaces. Salud Pública de México. 2021; 63(2):232-241.

21. Perski O, Szinay D, Corker E, Shahab L, West R, Michie S. Interventions to increase personal protective behaviours to limit the spread of respiratory viruses: A rapid evidence review and meta‐analysis. British Journal of Health Psychology. 2022; 27(1):215-264.

22. Saunders-Hastings P, Crispo JA, Sikora L, Krewski D. Effectiveness of personal protective measures in reducing pandemic influenza transmission: A systematic review and meta-analysis. Epidemics. 2017; 20:1-20.

23. Talic S, Shah S, Wild H, Gasevic D, Maharaj A, Ademi Z et al. Effectiveness of public health measures in reducing the incidence of covid-19, SARS-CoV-2 transmission, and covid-19 mortality: systematic review and meta-analysis. bmj. 2021; 375.

24. Veys K, Dockx K, Van Remoortel H, Vandekerckhove P, De Buck E. The effect of hand hygiene promotion programs during epidemics and pandemics of respiratory droplet-transmissible infections on health outcomes: a rapid systematic review. BMC public health. 2021; 21(1):1-11.

25. Wong VW, Cowling BJ, Aiello AE. Hand hygiene and risk of influenza virus infections in the community: a systematic review and meta-analysis. Epidemiology & Infection. 2014; 142(5):922-932.

26. Lange S, Barnard T, Naicker N. A scoping review to identify the type and effect of hand hygiene interventions on the reduction of infectious diseases (including COVID-19) in pre-school children. South African Journal of Child Health. 2021; 15(4):218-223.

27. Ford N, Holmer HK, Chou R, Villeneuve PJ, Baller A, Van Kerkhove M et al. Mask use in community settings in the context of COVID-19: A systematic review of ecological data. EClinicalMedicine. 2021; 38:101024.

**Not specific to the general public population (n=5)**

1. Adhikari SP, Meng S, Wu YJ, Mao YP, Ye RX, Wang QZ et al. Epidemiology, causes, clinical manifestation and diagnosis, prevention and control of coronavirus disease (COVID-19) during the early outbreak period: a scoping review. Infectious Diseases of Poverty. 2020; 9(1):29-29.

2. Fung IC, Cairncross S. Effectiveness of handwashing in preventing SARS: a review. Tropical medicine & international health : TM & IH. 2006; 11(11):1749-1758.

3. Cheng VC, Tai JW, Wong L, Chan JF, Li IW, To K et al. Prevention of nosocomial transmission of swine-origin pandemic influenza virus A/H1N1 by infection control bundle. Journal of Hospital Infection. 2010; 74(3):271-277.

4. Chung H, Kim EO, Kim S-H, Jung J. Risk of COVID-19 transmission from infected outpatients to healthcare workers in an outpatient clinic. Journal of Korean Medical Science. 2020; 35(50).

5. Ranjan P, Bhattacharya A, Chakrawarty A, Das R, Kumar A, Pandey S et al. Association between self-reported adherence to preventive practices and probability of turning COVID-19 positive: a cross-sectional analytical study. Cureus. 2020; 12(12).

**Area of interest not specific to hand hygiene (n=12)**

**Focus not specific to the effectiveness of hand hygiene (n=7)**

1. Maclntyre CR, Chughtai AA. Facemasks for the prevention of infection in healthcare and community settings. Bmj-British Medical Journal. 2015; 350:H694-H694.

2. Hoang VT, Gautret P. Infectious Diseases and Mass Gatherings. Current Infectious Disease Reports. 2018; 20(11).

3. Hoang V-T, Goumballa N, Dao T-L, Ly TDA, Ninove L, Ranque S et al. Respiratory and gastrointestinal infections at the 2017 Grand Magal de Touba, Senegal: A prospective cohort survey. Travel Medicine & Infectious Disease. 2019; 32:N.PAG-N.PAG.

4. Tang CS, Wong C-y. An outbreak of the severe acute respiratory syndrome: predictors of health behaviors and effect of community prevention measures in Hong Kong, China. American Journal of Public Health. 2003; 93(11):1887-1888.

5. France AM, Jackson M, Schrag S, Lynch M, Zimmerman C, Biggerstaff M et al. Household transmission of 2009 influenza A (H1N1) virus after a school-based outbreak in New York City, April–May 2009. The Journal of infectious diseases. 2010; 201(7):984-992.

6. Hirotsu N, Wada K, Oshitani H. Risk factors of household transmission of pandemic (H1N1) 2009 among patients treated with antivirals: a prospective study at a primary clinic in Japan. PloS one. 2012; 7(2):e31519.

7. Torner N, Morteruel M, Martínez A, Godoy P. Influenza (H1N1) 2009 outbreaks in educational settings. Catalonia. Revista Espanola de Salud Publica. 2011; 85(1):97-103.

**Hand hygiene being an element of multicomponent intervention (n=4)**

1. Lee VJ, Yap J, Cook AR, Chen MI, Tay JK, Barr I et al. Effectiveness of public health measures in mitigating pandemic influenza spread: a prospective sero-epidemiological cohort study. The Journal of Infectious Diseases. 2010; 202(9):1319-1326.

2. Mniszewski SM, Del Valle SY, Priedhorsky R, Hyman JM, Hickman KS. Understanding the Impact of Face Mask Usage Through Epidemic Simulation of Large Social Networks. In: *Theories and Simulations of Complex Social Systems. Volume 52*, edn. Edited by Dabbaghian V, Mago VK; 2014: 97-115.

3. Stebbins S, Stark JH, Vukotich Jr CJ. Compliance with a multilayered nonpharmaceutical intervention in an urban elementary school setting. Journal of Public Health Management and Practice. 2010; 16(4):316-324.

4. Suess T, Remschmidt C, Schink SB, Schweiger B, Nitsche A, Schroeder K et al. The role of facemasks and hand hygiene in the prevention of influenza transmission in households: results from a cluster randomised trial; Berlin, Germany, 2009-2011. Bmc Infectious Diseases. 2012; 12.

**Hand hygiene being a comparator to another intervention (n=1)**

1. Simmerman JM, Suntarattiwong P, Levy J, Jarman RG, Kaewchana S, Gibbons RV et al. Findings from a household randomized controlled trial of hand washing and face masks to reduce influenza transmission in Bangkok, Thailand. Influenza and Other Respiratory Viruses. 2011; 5(4):256-267.

**Outcomes not meeting eligibility criteria (n=71)**

**Outcomes not specific to respiratory viruses that cause pandemic or epidemics e.g. non-specific respiratory symptoms or not showing actual transmission or acquisition (n=32)**

1. Aledort JE, Lurie N, Wasserman J, Bozzette SA. Non-pharmaceutical public health interventions for pandemic influenza: an evaluation of the evidence base. BMC Public Health. 2007; 7(1):208.

2. Al-Jasser FS, Kabbash IA, AlMazroa MA, Memish ZA. Patterns of diseases and preventive measures among domestic hajjis from Central, Saudi Arabia. Saudi Medical Journal. 2012; 33(8):879-886.

3. Balaban V, Stauffer WM, Hammad A, Afgarshe M, Abd‐Alla M, Ahmed Q et al. Protective practices and respiratory illness among US travelers to the 2009 Hajj. Journal of Travel Medicine. 2012; 19(3):163-168.

4. Benkouiten S, Brouqui P, Gautret P. Non-pharmaceutical interventions for the prevention of respiratory tract infections during Hajj pilgrimage. Travel medicine and infectious disease. 2014; 12(5):429-442.

5. Biran A, Schmidt W, Varadharajan K, Rajaraman D, Kumar R, Gopalan B. Effect of a behaviour-change intervention on handwashing with soap in India (SuperAmma): a cluster-randomised trial Lancet Global Health. 2014; 2(3):e145–154.

6. Gautret P, Vu Hai V, Sani S, Doutchi M, Parola P, Brouqui P. Protective measures against acute respiratory symptoms in French pilgrims participating in the Hajj of 2009. Journal of Travel Medicine. 2011; 18(1):53-55.

7. Guinan M, McGuckin M, Ali Y. The effect of a comprehensive handwashing program on absenteeism in elementary schools. American Journal of Infection Control. 2002; 30(4):217-220.

8. Lee T, Jordan NN, Sanchez JL, Gaydos JC. Selected nonvaccine interventions to prevent infectious acute respiratory disease. American Journal of Preventive Medicine. 2005; 28(3):305-316.

9. Luby SP, Agboatwalla M, Feikin DR, Painter J, Billhimer W, Altaf A et al. Effect of handwashing on child health: a randomised controlled trial. The Lancet. 2005; 366(9481):225-233.

10. Sandora TJ, Taveras EM, Shih M-C, Resnick EA, Lee GM, Ross-Degnan D et al. A randomized, controlled trial of a multifaceted intervention including alcohol-based hand sanitizer and hand-hygiene education to reduce illness transmission in the home. Pediatrics. 2005; 116(3):587-594.

11. Sattar SA, Springthorpe VS, Tetro J, Vashon R, Keswick B. Hygienic hand antiseptics: should they not have activity and label claims against viruses? Am J Infect Control. 2002; 30(6):355-372.

12. Tamimi AH, Carlino S, Edmonds S, Gerba CP. Impact of an Alcohol-Based Hand Sanitizer Intervention on the Spread of Viruses in Homes. Food and Environmental Virology. 2014; 6(2):140-144.

13. White C, Kolble R, Carlson R, Lipson N. The impact of a health campaign on hand hygiene and upper respiratory illness among college students living in residence halls. Journal of American College Health. 2005; 53(4):175-181.

14. White C, Kolble R, Carlson R, Lipson N, Dolan M, Ali Y et al. The effect of hand hygiene on illness rate among students in university residence halls. American Journal of Infection Control. 2003; 31(6):364-370.

15. Al-Ansari F, Mirzaei M, Al-Ansari B, Al-Ansari MB, Abdulzahra MS, Rashid H et al. Health risks, preventive behaviours and respiratory illnesses at the 2019 arbaeen: Implications for covid-19 and other pandemics. International Journal of Environmental Research and Public Health. 2021; 18(6):3287.

16. Alqahtani AS, Alsharif SA, Garnan MA, Tashani M, BinDhim NF, Heywood AE et al. The Impact of Receiving Pretravel Health Advice on the Prevention of Hajj-Related Illnesses Among Australian Pilgrims: Cohort Study. JMIR Public Health and Surveillance. 2020; 6(3):e10959.

17. Azor-Martínez E, Gonzalez-Jimenez Y, Seijas-Vazquez ML, Cobos-Carrascosa E, Santisteban-Martínez J, Martínez-López JM et al. The impact of common infections on school absenteeism during an academic year. American journal of infection control. 2014; 42(6):632-637.

18. Azor-Martinez E, Yui-Hifume R, Muñoz-Vico FJ, Jimenez-Noguera E, Strizzi JM, Martinez-Martinez I et al. Effectiveness of a hand hygiene program at child care centers: a cluster randomized trial. Pediatrics. 2018; 142(5).

19. Bara’a HMI, Nori MMM, Abdallah WS, Ali SM. Coronavirus 2019-like illness and public adherence to preventive measures, Sudan 2020. Journal of preventive medicine and hygiene. 2021; 62(2):E305.

20. Bowen A, Ma H, Ou J, Billhimer W, Long T, Mintz E et al. A cluster-randomized controlled trial evaluating the effect of a handwashing-promotion program in Chinese primary schools. The American journal of tropical medicine and hygiene. 2007; 76(6):1166-1173.

21. Freeman MC, Greene LE, Dreibelbis R, Saboori S, Muga R, Brumback B et al. Assessing the impact of a school‐based water treatment, hygiene and sanitation programme on pupil absence in Nyanza Province, Kenya: a cluster‐randomized trial. Tropical medicine & international health. 2012; 17(3):380-391.

22. Herrera-Añazco P, Urrunaga-Pastor D, Benites-Zapata VA, Bendezu-Quispe G, Toro-Huamanchumo CJ, Hernandez AV. COVID-19 symptomatology and compliance with community mitigation strategies in Latin America early during the COVID-19 pandemic. Preventive medicine reports. 2022; 25:101665.

23. Little P, Stuart B, Hobbs FR, Moore M, Barnett J, Popoola D et al. An internet-delivered handwashing intervention to modify influenza-like illness and respiratory infection transmission (PRIMIT): a primary care randomised trial. The Lancet. 2015; 386(10004):1631-1639.

24. Lopez-Quintero C, Freeman P, Neumark Y. Hand washing among school children in Bogota, Colombia. American Journal of public health. 2009; 99(1):94-101.

25. Loustalot F, Silk BJ, Gaither A, Shim T, Lamias M, Dawood F et al. Household transmission of 2009 pandemic influenza A (H1N1) and nonpharmaceutical interventions among households of high school students in San Antonio, Texas. Clinical Infectious Diseases. 2011; 52(suppl_1):S146-S153.

26. Masai AN. Practice of COVID-19 preventive measures and risk of acute respiratory infections: a longitudinal study in students from 95 countries. International Journal of Infectious Diseases. 2021; 113:168-174.

27. Merk H, Kühlmann-Berenzon S, Linde A, Nyrén O. Associations of hand-washing frequency with incidence of acute respiratory tract infection and influenza-like illness in adults: a population-based study in Sweden. BMC infectious diseases. 2014; 14(1):1-8.

28. Nasreen S, Azziz‐Baumgartner E, Gurley E, Winch P, Unicomb L, Sharker M et al. Prevalent high‐risk respiratory hygiene practices in urban and rural Bangladesh. Tropical Medicine & International Health. 2010; 15(6):762-771.

29. Nicholson JA, Naeeni M, Hoptroff M, Matheson JR, Roberts AJ, Taylor D et al. An investigation of the effects of a hand washing intervention on health outcomes and school absence using a randomised trial in Indian urban communities. Tropical Medicine & International Health. 2014; 19(3):284-292.

30. Raciborski F, Jankowski M, Gujski M, Pinkas J, Samel-Kowalik P, Zaczyński A et al. Prevention of SARS-CoV-2 infection among police officers in Poland—implications for public health policies. International journal of environmental research and public health. 2020; 17(23):9072.

31. Remschmidt C, Stöcker P, an der Heiden M, Suess T, Luchtenberg M, Schink SB et al. Preventable and non‐preventable risk factors for influenza transmission and hygiene behavior in German influenza households, pandemic season (H1N1) 2009/2010. Influenza and other respiratory viruses. 2013; 7(3):418-425.

32. White CG, Shinder FS, Shinder AL, Dyer DL. Reduction of illness absenteeism in elementary schools using an alcohol-free instant hand sanitizer. The Journal of School Nursing. 2001; 17(5):258-265.

**Outcomes other than acquisition or transmission of respiratory infections (n=39)**

1. Al-Hazmi A, Gosadi I, Somily A, Alsubaie S, Bin Saeed A. Knowledge, attitude and practice of secondary schools and university students toward Middle East Respiratory Syndrome epidemic in Saudi Arabia: A cross-sectional study. Saudi Journal of Biological Sciences. 2018; 25(3):572-577.

2. Al-Mohrej OA, Al-Shirian SD, Al-Otaibi SK, Tamim HM, Masuadi EM, Fakhoury HM. Is the Saudi public aware of Middle East respiratory syndrome? Journal of Infection and Public Health. 2016; 9(3):259-266.

3. Alzoubi H, Alnawaiseh N, Al-Mnayyis A, Abu-Lubad M, Aqel A, Al-Shagahin H. COVID-19-Knowledge, Attitude and Practice among Medical and Non-Medical University Students in Jordan. Journal of Pure and Applied Microbiology. 2020; 14(1):17-24.

4. Chan EYY, Cheng CKY, Tam G, Huang Z, Lee P. Knowledge, attitudes, and practices of Hong Kong population towards human A/H7N9 influenza pandemic preparedness, China, 2014. Bmc Public Health. 2015; 15:943-943.

5. Chaung S. The Effect of Hand Washing Procedure Poster on the Hand Washing Behaviors. The journal of Convergence on Culture Technology. 2018; 4(3):269-274.

6. Chen X, Ran L, Liu Q, Hu Q, Du X, Tan X. Hand Hygiene, Mask-Wearing Behaviors and Its Associated Factors during the COVID-19 Epidemic: A Cross-Sectional Study among Primary School Students in Wuhan, China. International journal of environmental research and public health. 2020; 17(8).

7. Chen YC, Chiang LC. Effectiveness of hand‐washing teaching programs for families of children in paediatric intensive care units. Journal of Clinical Nursing. 2007; 16(6):1173-1179.

8. Freiman AJ, Montgomery JP, Green JJ, Thomas DL, Kleiner AM, Boulton ML. Did H1N1 Influenza Prevention Messages Reach the Vulnerable Population Along the Mississippi Gulf Coast? Journal of Public Health Management and Practice. 2011; 17(1):52-58.

9. Fung IC, Cairncross S. How often do you wash your hands? a review of studies of hand-washing practices in the community during and after the SARS outbreak in 2003. International Journal of Environmental Health Research. 2007; 17(3):161-183.

10. Gutierrez-Dona B, Renner B, Reuter T, Giese H, Schubring D. Health behavior education, e-research and a (H1N1) influenza (Swine Flu): bridging the gap between intentions and health behavior change. In: *4th World Conference on Educational Sciences. Volume 46*, edn. Edited by Baskan GA, Ozdamli F, Kanbul S, Ozcan D; 2012: 2782-2795.

11. Hammond B, Ali Y, Fendler E, Dolan M, Donovan S. Effect of hand sanitizer use on elementary school absenteeism. American Journal of Infection Control. 2000; 28(5):340-346.

12. Hübner N-O, Hübner C, Wodny M, Kampf G, Kramer A. Effectiveness of alcohol-based hand disinfectants in a public administration: impact on health and work performance related to acute respiratory symptoms and diarrhoea. BMC Infectious Diseases. 2010; 10(1):250.

13. Jang WM, Cho S, Jang DH, Kim UN, Jung H, Lee JY et al. Preventive Behavioral Responses to the 2015 Middle East Respiratory Syndrome Coronavirus Outbreak in Korea. International Journal of Environmental Research and Public Health. 2019; 16(12):2161-2161.

14. Lau JTF, Griffiths S, Au DWH, Choi KC. Changes in knowledge, perceptions, preventive behaviours and psychological responses in the pre-community outbreak phase of the H1N1 epidemic. Epidemiology and Infection. 2011; 139(1):80-90.

15. Lau JTF, Griffiths S, Choi KC, Tsui HY. Widespread public misconception in the early phase of the H1N1 influenza epidemic. Journal of Infection. 2009; 59(2):122-127.

16. Lau JTF, Kim JH, Tsui HY, Griffiths S. Anticipated and current preventive behaviors in response to an anticipated human-to-human H5N1 epidemic in the Hong Kong Chinese general population. Bmc Infectious Diseases. 2007; 7.

17. Lau JTF, Tsui HY, Kim JH, Chan PKS, Griffiths S. Monitoring of perceptions, anticipated behavioral, and psychological responses related to H5N1 influenza. Infection. 2010; 38(4):275-283.

18. Lau JTF, Yang X, Tsui H, Kim JH. Monitoring community responses to the SARS epidemic in Hong Kong: from day 10 to day 62. Journal of Epidemiology and Community Health. 2003; 57(11):864-870.

19. Lau JTF, Yang X, Tsui HY, Pang E. SARS related preventive and risk behaviours practised by Hong Kong-mainland China cross border travellers during the outbreak of the SARS epidemic in Hong Kong. Journal of Epidemiology & Community Health. 2004; 58(12):988-996.

20. Lee M, Ju Y, You M. The Effects of Social Determinants on Public Health Emergency Preparedness Mediated by Health Communication: The 2015 MERS Outbreak in South Korea. Health Communication. 2019.

21. Lee SY, Yang HJ, Kim G, Cheong H-K, Youl CB. Preventive behaviors by the level of perceived infection sensitivity during the Korea outbreak of Middle East Respiratory Syndrome in 2015. Korean Journal of Epidemiology. 2016; 38:1-10.

22. Lee-Baggley D, DeLongis A, Voorhoeave P, Greenglass E. Coping with the threat of severe acute respiratory syndrome: Role of threat appraisals and coping responses in health behaviors. Asian Journal of Social Psychology. 2004; 7(1):9-23.

23. Liao QY, Cowling BJ, Lam WWT, Fielding R. The Influence of Social-Cognitive Factors on Personal Hygiene Practices to Protect Against Influenzas: Using Modelling to Compare Avian A/H5N1 and 2009 Pandemic A/H1N1 Influenzas in Hong Kong. International Journal of Behavioral Medicine. 2011; 18(2):93-104.

24. Meadows E, Le Saux N. A systematic review of the effectiveness of antimicrobial rinse-free hand sanitizers for prevention of illness-related absenteeism in elementary school children. BMC Public Health. 2004; 4(1):50.

25. Min JW, Chang Y-S. An experience of personal hygiene education and hand-washing practices among adolescents in the Korean youth risk behavior web-based survey. The Journal of Korean Society for School & Community Health Education. 2014; 15(1):31-43.

26. Morrison LG, Yardley L. What infection control measures will people carry out to reduce transmission of pandemic influenza? A focus group study. Bmc Public Health. 2009; 9:258-258.

27. Morton JL, Schultz AA. Healthy hands: use of alcohol gel as an adjunct to handwashing in elementary school children. The Journal of School Nursing. 2004; 20(3):161-167.

28. Park JH, Cheong HK, Son DY, Kim SU, Ha CM. Perceptions and behaviors related to hand hygiene for the prevention of H1N1 influenza transmission among Korean university students during the peak pandemic period. Bmc Infectious Diseases. 2010; 10.

29. Rubin GJ, Amlot R, Page L, Wessely S. Public perceptions, anxiety, and behaviour change in relation to the swine flu outbreak: cross sectional telephone survey. Bmj-British Medical Journal. 2009; 339:B2651-B2651.

30. Seale H, Mak JPI, Razee H, MacIntyre CR. Examining the knowledge, attitudes and practices of domestic and international university students towards seasonal and pandemic influenza. Bmc Public Health. 2012; 12:307-307.

31. Tan XD, Li SY, Wang CH, Chen XQ, Wu XM. Severe Acute Respiratory Syndrome epidemic and change of people's health behavior in China. Health Education Research. 2004; 19(5):576-580.

32. Wong JSW, Lee JKF. The Common Missed Handwashing Instances and Areas after 15 Years of Hand-Hygiene Education. Journal of Environmental & Public Health. 2019:1-7.

33. Yang J, Park E-C, Lee SA, Lee SG. Associations Between Hand Hygiene Education and Self-Reported Hand-Washing Behaviors Among Korean Adults During MERS-CoV Outbreak. Health Education & Behavior. 2019; 46(1):157-164.

34. Yardley L, Miller S, Schlotz W, Little P. Evaluation of a Web-Based Intervention to Promote Hand Hygiene: Exploratory Randomized Controlled Trial. Journal of Medical Internet Research. 2011; 13(4):E107-E107.

35. Bagi HM, Soleimanpour M, Abdollahi F, Soleimanpour H. Evaluation of clinical outcomes of patients with mild symptoms of coronavirus disease 2019 (COVID-19) discharged from the emergency department. PloS one. 2021; 16(10):e0258697.

36. Correa JC, Pinto D, Salas LA, Camacho JC, Rondón M, Quintero J. A cluster-randomized controlled trial of handrubs for prevention of infectious diseases among children in Colombia. Revista Panamericana de Salud Pública. 2012; 31:476-484.

37. Hommes F, van Loon W, Thielecke M, Abramovich I, Lieber S, Hammerich R et al. SARS-CoV-2 infection, risk perception, behaviour and preventive measures at schools in Berlin, Germany, during the early post-lockdown phase: a cross-sectional study. International Journal of Environmental Research and Public Health. 2021; 18(5):2739.

38. Lennell A, Kühlmann‐Berenzon S, Geli P, Hedin K, Petersson C, Cars O et al. Alcohol‐based hand‐disinfection reduced children's absence from Swedish day care centers. Acta Paediatrica. 2008; 97(12):1672-1680.

39. Savolainen-Kopra C, Haapakoski J, Peltola PA, Ziegler T, Korpela T, Anttila P et al. Hand washing with soap and water together with behavioural recommendations prevents infections in common work environment: an open cluster-randomized trial. Trials. 2012; 13(1):1-11.

**Other reasons (n=9)**

**Self-reported infection outcomes (n=2)**

1. Sharif N, Alzahrani KJ, Ahmed SN, Opu RR, Ahmed N, Talukder A et al. Protective measures are associated with the reduction of transmission of COVID-19 in Bangladesh: A nationwide cross-sectional study. Plos one. 2021; 16(11):e0260287.

2. Xu H, Gan Y, Zheng D, Wu B, Zhu X, Xu C et al. Relationship between COVID-19 infection and risk perception, knowledge, attitude, and four nonpharmaceutical interventions during the late period of the COVID-19 epidemic in China: online cross-sectional survey of 8158 adults. Journal of medical Internet research. 2020; 22(11):e21372.

**Cases confirmed by serology test (n=3)**

1. Dupraz J, Butty A, Duperrex O, Estoppey S, Faivre V, Thabard J et al. Prevalence of SARS-CoV-2 in household members and other close contacts of COVID-19 cases: a serologic study in canton of Vaud, Switzerland. In: *Open forum infectious diseases: 2021*: Oxford University Press US; 2021: ofab149.

2. Li T, Liu Y, Di B, Wang M, Shen J, Zhang Y et al. Epidemiological investigation of an outbreak of pandemic influenza A (H1N1) 2009 in a boarding school: serological analysis of 1570 cases. Journal of Clinical Virology. 2011; 50(3):235-239.

3. Liu W, Yang P, Duan W, Wang X, Zhang Y, Wang Q. Factors associated with seropositivity of 2009 H1N1 influenza in Beijing, China. Clin Infect Dis. 2010; 51(2):251-252.

**Data not collected at individual level (n=1)**

1. Suh HH, Meehan J, Blaisdell L, Browne L. Non-pharmaceutical interventions and COVID-19 cases in US summer camps: results from an American Camp Association survey. J Epidemiol Community Health. 2022; 76(4):327-334.

**Did not investigate for a relationship between hand hygiene and acquisition or transmission of infections of interest (n=1)**

1. Juhn YJ, Wi C-I, Ryu E, Sampathkumar P, Takahashi PY, Yao JD et al. Adherence to Public Health Measures Mitigates the Risk of COVID-19 Infection in Older Adults: A Community-Based Study. In: *Mayo Clinic Proceedings: 2021*: Elsevier; 2021: 912-920.

**Population-based study (n=2)**

1. Ahmed MS, Yunus FM. Trend of COVID-19 spreads and status of household handwashing practice and its determinants in Bangladesh–situation analysis using national representative data. International Journal of Environmental Health Research. 2022; 32(5):1002-1010.

2. Skolmowska D, Głąbska D, Guzek D. Hand hygiene behaviors in a representative sample of Polish adolescents in regions stratified by COVID-19 morbidity and by confounding variables (PLACE-19 Study): Is there any association? Pathogens. 2020; 9(12):1011.
