# Supplementary material for: The effectiveness of hand hygiene interventions for preventing community transmission or acquisition of novel coronavirus or influenza infections: a systematic review
Source: BMC Public Health. 2022 Jul 2;22:1283. doi: 10.1186/s12889-022-13667-y (PMC9250256; doi:10.1186/s12889-022-13667-y)
Supplement: Supplementary file 3 — Additional file 3. Quality Assessment Tables (Table A. Risk of bias of randomised controlled trials; Table B. Quality assessment of case-controlled studies; Table C. Quality assessment of cross-sectional studies; Table D. Quality assessment of cohort studies). [file 12889_2022_13667_MOESM3_ESM.docx]

# Additional file 3. Quality Assessment Tables

Table A. Risk of bias of randomised controlled trials

| Study ID | Was the allocation sequence adequately generated? | Was the allocation adequately concealed? | Were baseline outcome measurements similar? | Were baseline characteristics similar? | Were incomplete outcome data adequately addressed? | Was knowledge of the allocated interventions adequately prevented during the study? | Was the study adequately protected against contamination? | Are reports of the study free of suggestion of selective outcome reporting? | Was the study apparently free of other problems that could put it at a high risk of bias? | **Overall risk of bias** |
| --- | --- | --- | --- | --- | --- | --- | --- | --- | --- | --- |
| Biswas et al. (2019)[1] | L | L | U | H | U | L | L | L | H | **High** |
| Cowling et al. (2009)[2] | L | L | L | L | L | L | H | L | L | **High** |
| Larson et al. (2010)[3] | U | U | U | H | L | L | U | L | H | **High** |
| Ram et al. (2015)[4] | U | U | U | U | L | L | U | L | H | **High** |
| Stebbins et al. (2011)[5] | L | L | U | H | U | L | L | L | H | **High** |
| Talaat et al. (2011)[6] | L | L | L | U | L | L | L | L | L | **Unclear** |

H=high risk; L=low risk; U=unclear risk

Table B. Quality assessment of case-controlled studies

| Study ID | Were the groups comparable other than the presence of disease in cases or the absence of disease in controls? | Were cases and controls matched appropriately? | Were the same criteria used for identification of cases and controls? | Was exposure measured in a standard, valid and reliable way? | Was exposure measured in the same way for cases and controls? | Were confounding factors identified? | Were strategies to deal with confounding factors stated? | Were outcomes assessed in a standard, valid and reliable way for cases and controls? | Was the exposure period of interest long enough to be meaningful? | Was appropriate statistical analysis used? | **Overall risk of bias** |
| --- | --- | --- | --- | --- | --- | --- | --- | --- | --- | --- | --- |
| Castilla et al. (2013)[7] | Y | Y | Y | U | Y | Y | Y | N | Y | Y | **High** |
| Doshi et al. (2015)[8] | Y | Y | N | N | U | Y | Y | N | N | U | **High** |
| Doung-ngern et al. (2020)[9] | N | U | N | U | Y | Y | Y | N | N | U | **High** |
| Godoy et al. (2012)[10] | Y | Y | Y | U | Y | Y | Y | N | Y | Y | **High** |
| Lau et al. (2004)[11] | Y | Y | Y | U | Y | Y | Y | Y | Y | U | **Unclear** |
| Lio et al. (2021)[12] | N | U | U | N | U | Y | Y | U | Y | U | **High** |
| Speaker et al. (2021)[13] | Y | Y | U | U | N | Y | N | U | N | U | **High** |
| Torner et al. (2015)[14] | Y | Y | U | U | U | Y | Y | U | Y | U | **Unclear** |
| Wu et al. (2004)[15] | Y | Y | Y | U | N | Y | Y | U | Y | Y | **High** |
| Zhang et al. (2013)[16] | Y | Y | Y | U | U | Y | Y | Y | U | U | **Unclear** |

Y=yes; N=no; U=unclear

Table C. Quality assessment of cross-sectional studies

| Study ID | Were the criteria for inclusion in the sample clearly defined? | Were the study subjects and the setting described in detail? | Was the exposure measured in a valid and reliable way? | Were objective, standard criteria used for measurement of the condition? | Were confounding factors identified? | Were strategies to deal with confounding factors stated? | Were the outcomes measured in a valid and reliable way? | Was appropriate statistical analysis used? | **Overall risk of bias** |
| --- | --- | --- | --- | --- | --- | --- | --- | --- | --- |
| Abd (2021)[17] | U | Y | U | U | Y | N | U | N | **High** |
| Badri et al. (2021)[18] | Y | Y | Y | Y | U | Y | Y | U | **Unclear** |
| Karout et al. (2020)[19] | Y | Y | U | Y | Y | N | Y | N | **High** |
| Wilson-Clark et al. (2006)[20] | Y | Y | Y | Y | Y | Y | Y | Y | **Low** |

Y=yes; N=no; U=unclear

Table D. Quality assessment of cohort studies

| Study ID | Were the two groups similar and recruited from the same population? | Were the exposures measured similarly to assign people to both exposed and unexposed groups? | Was the exposure measured in a valid and reliable way? | Were confounding factors identified? | Were strategies to deal with confounding factors stated? | Were the groups/participants free of the outcome at the start of the study (or at the moment of exposure)? | Were the outcomes measured in a valid and reliable way? | Was the follow up time reported and sufficient to be long enough for outcomes to occur? | Was follow up complete, and if not, were the reasons to loss to follow up described and explored? | Were strategies to address incomplete follow up utilized? | Was appropriate statistical analysis used? | **Overall risk of bias** |
| --- | --- | --- | --- | --- | --- | --- | --- | --- | --- | --- | --- | --- |
| Liu et al. (2021)[21] | U | Y | U | N | N | U | Y | Y | Y | Y | N | **High** |
| Xie et al. (2021)[22] | Y | Y | U | Y | Y | U | Y | U | Y | Y | U | **Unclear** |

Y=yes; N=no; U=unclear

## References

1. Biswas D, Ahmed M, Roguski K, Ghosh PK, Parveen S, Nizame FA et al. Effectiveness of a behavior change intervention with hand sanitizer use and respiratory hygiene in reducing laboratory-confirmed influenza among schoolchildren in Bangladesh: a cluster randomized controlled trial. Am J Trop Med Hyg. 2019; 101(6):1446.

2. Cowling BJ, Chan KH, Fang VJ, Cheng CKY, Fung ROP, Wai W et al. Facemasks and hand hygiene to prevent influenza transmission in households: a cluster randomized trial. Ann Intern Med. 2009; 151(7):437-466.

3. Larson EL, Ferng Y-H, Wong-McLoughlin J, Wang S, Haber M, Morse SS. Impact of non-pharmaceutical interventions on URIs and influenza in crowded, urban households. Public Health Rep. 2010; 125(2):178-191.

4. Ram PK, DiVita MA, Khatun-e-Jannat K, Islam M, Krytus K, Cercone E et al. Impact of intensive handwashing promotion on secondary household influenza-like illness in rural bangladesh: findings from a randomized controlled trial. PloS One. 2015; 10(6):e0125200.

5. Stebbins S, Cummings DA, Stark JH, Vukotich C, Mitruka K, Thompson W et al. Reduction in the incidence of influenza A but not influenza B associated with use of hand sanitizer and cough hygiene in schools: a randomized controlled trial. Pediatr Infect Dis J. 2011; 30(11):921.

6. Talaat M, Afifi S, Dueger E, El-Ashry N, Marfin A, Kandeel A et al. Effects of hand hygiene campaigns on incidence of laboratory-confirmed influenza and absenteeism in schoolchildren, Cairo, Egypt. Emerg Infect Dis. 2011; 17(4):619-625.

7. Castilla J, Godoy P, Dominguez A, Martin V, Delgado-Rodriguez M, Martinez-Baz I et al. Risk factors and effectiveness of preventive measures against influenza in the community. Influenza Other Respir Viruses. 2013; 7(2):177-183.

8. Doshi S, Silk BJ, Dutt D, Ahmed M, Cohen AL, Taylor TH et al. Household‐level risk factors for influenza among young children in Dhaka, Bangladesh: a case–control study. Trop Med Int Health. 2015; 20(6):719-729.

9. Doung-Ngern P, Suphanchaimat R, Panjangampatthana A, Janekrongtham C, Ruampoom D, Daochaeng N et al. Case-Control Study of Use of Personal Protective Measures and Risk for SARS-CoV 2 Infection, Thailand. Emerg Infect Dis. 2020; 26(11):2607-2616.

10. Godoy P, Castilla J, Delgado-Rodriguez M, Martin V, Soldevila N, Alonso J et al. Effectiveness of hand hygiene and provision of information in preventing influenza cases requiring hospitalization. Prevent Med. 2012; 54(6):434-439.

11. Lau JTF, Tsui H, Lau M, Yang XL. SARS transmission, risk factors, and prevention in Hong Kong. Emerg Infect Dis. 2004; 10(4):587-592.

12. Lio CF, Cheong HH, Lei CI, Lo IL, Yao L, Lam C et al. Effectiveness of personal protective health behaviour against COVID-19. BMC Public Health. 2021; 21(1).

13. Speaker SL, Doherty CM, Pfoh E, Dunn A, Hair B, Daboul L et al. Social Behaviors Associated With a Positive COVID-19 Test Result. Cureus. 2021; 13(2).

14. Torner N, Soldevila N, Garcia JJ, Launes C, Godoy P, Castilla J et al. Effectiveness of non-pharmaceutical measures in preventing pediatric influenza: a case–control study. BMC Public Health. 2015; 15(1):1-8.

15. Wu J, Xu F, Zhou W, Feikin DR, Lin C-Y, He X et al. Risk factors for SARS among persons without known contact with SARS patients, Beijing, China. Emerg Infect Dis. 2004; 10(2):210.

16. Zhang D, Liu W, Yang P, Zhang Y, Li X, Germ KE et al. Factors associated with household transmission of pandemic (H1N1) 2009 among self-quarantined patients in Beijing, China. PloS One. 2013; 8(10):e77873.

17. Abd RK. Prevalence and Risk Factors for COVID-19 Infection at Thi- Qar Governorate, Southern Iraq. Lat Am J Pharm. 2021; 40(SI):248-253.

18. Badri S, Sardá V, Moncada JS, Merçon M, Rezai K, Weinstein RA et al. Disparities and Temporal Trends in COVID-19 Exposures and Mitigating Behaviors Among Black and Hispanic Adults in an Urban Setting. JAMA Netw Open. 2021; 4(9):e2125187-e2125187.

19. Karout L, Serwat A, El Mais H, Kassab M, Khalid F, Mercedes BR. COVID-19 prevalence, risk perceptions, and preventive behavior in asymptomatic Latino population: A cross-sectional study. Cureus. 2020; 12(9):e10707.

20. Wilson-Clark SD, Deeks SL, Gournis E, Hay K, Bondy S, Kennedy E et al. Household transmission of SARS, 2003. CMAJ. 2006; 175(10):1219-1223.

21. Liu PY, Gragnani CM, Timmerman J, Newhouse CN, Soto G, Lopez L et al. Pediatric Household Transmission of Severe Acute Respiratory Coronavirus-2 Infection-Los Angeles County, December 2020 to February 2021. Pediatr Infect Dis J. 2021; 40(10):e379-e381.

22. Xie W, Chen Z, Wang Q, Song M, Cao Y, Wang L et al. Infection and disease spectrum in individuals with household exposure to SARS-CoV-2: A family cluster cohort study. J Med Virol. 2021; 93(5):3033-3046.
